# Transcriptome Analysis and Gene Expression Profiling of Abortive and Developing Ovules during Fruit Development in Hazelnut

**DOI:** 10.1371/journal.pone.0122072

**Published:** 2015-04-02

**Authors:** Yunqing Cheng, Jianfeng Liu, Huidi Zhang, Ju Wang, Yixin Zhao, Wanting Geng

**Affiliations:** College of Life Sciences, Jilin Normal University, Siping, Jilin Province 136000, China; Nazarbayev University, KAZAKHSTAN

## Abstract

**Background:**

A high ratio of blank fruit in hazelnut (*Corylus heterophylla* Fisch) is a very common phenomenon that causes serious yield losses in northeast China. The development of blank fruit in the *Corylus* genus is known to be associated with embryo abortion. However, little is known about the molecular mechanisms responsible for embryo abortion during the nut development stage. Genomic information for *C*. *heterophylla* Fisch is not available; therefore, data related to transcriptome and gene expression profiling of developing and abortive ovules are needed.

**Methodology/Principal Findings:**

In this study, de novo transcriptome sequencing and RNA-seq analysis were conducted using short-read sequencing technology (Illumina HiSeq 2000). The results of the transcriptome assembly analysis revealed genetic information that was associated with the fruit development stage. Two digital gene expression libraries were constructed, one for a full (normally developing) ovule and one for an empty (abortive) ovule. Transcriptome sequencing and assembly results revealed 55,353 unigenes, including 18,751 clusters and 36,602 singletons. These results were annotated using the public databases NR, NT, Swiss-Prot, KEGG, COG, and GO. Using digital gene expression profiling, gene expression differences in developing and abortive ovules were identified. A total of 1,637 and 715 unigenes were significantly upregulated and downregulated, respectively, in abortive ovules, compared with developing ovules. Quantitative real-time polymerase chain reaction analysis was used in order to verify the differential expression of some genes.

**Conclusions/Significance:**

The transcriptome and digital gene expression profiling data of normally developing and abortive ovules in hazelnut provide exhaustive information that will improve our understanding of the molecular mechanisms of abortive ovule formation in hazelnut.

## Introduction

Hazelnut (*Corylus* L. spp.) is an edible nut crop that has great economic value. Its kernel is an important ingredient that is widely used in the production of dairy products, baked goods, chocolates, and other confectionery products. The unsaturated fatty acids in the kernel are reported to provide human health benefits by lowering cholesterol levels in blood and controlling the adverse effects of hypertension [1, 2]. Due to the economic and ecological importance of hazelnut cultivation in northeast China, it is an important option for farmers who wish to increase their incomes in mountainous areas [3, 4].

*C*. *heterophylla* Fisch. ex Besser and its hybrids with European hazelnut (*C*. *heterophylla* × *C*. *avellana*) are the most important *Corylus* varieties that are cultivated in northeast China, and their excellent resistance to cold will ensure their continued dominance of hazelnut production in China in the short term. However, excessive empty fruit formation of *C*. *heterophylla* causes serious yield losses [3]. A number of studies have suggested that self-incompatibility is associated with a higher frequency of blanks, nuts that contain no edible kernel [5, 6]. In a previous study, empty fruit formation of *C*. *heterophylla* Fisch was verified to be closely associated with embryo abortion, using comparisons of morphological anatomy in normally developing nuts with empty nuts [3]. However, the molecular regulation mechanisms of embryo abortion in hazelnut remain unknown.

In recent years, effective next-generation genome-wide gene profiling techniques have provided fascinating opportunities to survey the coding sequences of a total genome [7]. For example, Illumina sequencing technology provides millions of sequence reads from a single instrument run, and the assembled unigenes may be mapped to a reference transcriptome profile to obtain their molecular annotations. Despite the rapidly increasing amount of sequencing data, expressed sequence tags (ESTs) of *C*. *avellana* remain the only available large-scale sequencing data for the *Corylus* genus [8]. Therefore, data from transcriptome and expression profiling of developing and abortive ovules are needed in order to understand the molecular mechanisms regulating ovule abortion in hazelnut.

In this study, we constructed a total pooled RNA library of developing and abortive ovules. De novo transcriptome Illumina sequencing of the pooled RNA library generated more than 4 billion nucleotides of high-quality RNA sequences. Due to the lack of genome information for hazelnut, 55,353 of the unigenes acquired provided necessary and useful reference sequences after transcriptome assembly and annotation analysis. Further transcriptome RNA-seq (quantification) analyses were carried out to identify differentially expressed genes (DEGs) that were associated with ovule and embryo development and to determine their possible functions. The assembled, annotated transcriptome sequences and gene expression profiles provide useful information that can help identify genes involved in embryo abortion during the fruit development stage.

## Materials and Methods

### Materials

Fruit from young hazelnut trees was sampled from a hazelnut orchard (43°07′06″N, 124°28′39″E) near Siping, Jilin province, China. No specific permission was required in order to work in this location; also, the experiment did not involve endangered or protected species in China. In early spring, pollen from the cultivar ‘Dawei’ was collected from the orchard and air-dried using the method described by Liu *et al*. [3]. At the same time, more than 600 pistillate inflorescences of *C*. *heterophylla* Fisch were randomly selected and bagged. On April 20, hand pollination was performed. Starting on June 20, fertilized ovules began to grow rapidly, resulting in an obvious size difference between developing ovules and abortive ovules. Young fruits were sampled three times at 15-day intervals after June 20. The samples were stored on ice until dissection, at which time the ovules from each fruit were carefully dissected. To obtain complete gene-expression data from ovules during the stages of fruit development, the sampled fruits were further dissected to determine whether the ovules were abortive. Developing ovules had diameters ranging from about 10 mm to more than 100 mm, and about 50 developing ovules in 50 fruits were collected. Abortive ovules had diameters of less than 3 mm during the fruit development stage and about 400 ovules were collected in 200 fruits. The developing and abortive ovules were immediately frozen in liquid nitrogen and stored at −80°C for RNA extraction.

### cDNA library preparation and Illumina sequencing for transcriptome analysis

Total RNA of the ovule samples was extracted using the RNA Easyspin Isolation System (Aidlab Biotech, Beijing, China), and RNase-free DNase I was used to eliminate the residual genomic DNA in the raw RNA extract, according to the manufacturer’s protocol (Promega, Beijing, China), with minor revisions. The concentration and integrity of total RNA were measured according to spectrophotometer analysis and using denaturing agarose gel electrophoresis. To obtain complete gene expression information, data from developing and abortive ovules at three equivalent stages were pooled, and these samples were used for transcriptome analysis.

After the pooled RNA samples were treated with DNase I, magnetic beads with Oligo (dT) were used to isolate mRNA. The mRNA was fragmented into short segments (about 200 bp) by mixing with the fragmentation buffer. Next, the first strand of cDNA was synthesized by using a random hexamer primer. Buffer, dNTPs, RNAase H, and DNA polymerase I were added to synthesize the second strand. After end repair was performed and a 3’-end single nucleotide A was added, the short fragments were connected using adapters. The fragments were enriched by polymerase chain reaction (PCR) amplification and purified to create cDNA libraries. The Agilent 2100 Bioanalyzer and the ABI StepOnePlus Real-Time PCR System were used respectively to quantify and qualify the sample libraries. Finally, the cDNA libraries were sequenced using HiSeq 2000 Sequencing System (Illumina, Inc., USA). All raw transcriptome data was deposited in SRA (NCBI BioSample Accessions Number: SAMN03152274, SAMN03152275).

Transcriptome de novo assembly was carried out with the short-read assembly program Trinity [9]. The resulting sequences were considered unigenes. The removal of redundant sequences and further splicing of the assembled unigenes from each sample were carried out using the software program TGICL [10], after which sequence clustering software was used to acquire non-redundant unigenes for as long as possible. BLASTX alignments (E-value < 10^−5^) between unigenes and protein databases, including NR, Swiss-Prot, the Kyoto Encyclopedia of Genes and Genomes (KEGG), and COG, were performed, and the best aligned results were used to determine the sequence direction of unigenes. If the results of different databases conflicted with one another, a priority order of NR, Swiss-Prot, KEGG, and COG was followed in order to decide the sequence direction of unigenes. When a unigene did not align with sequences in any of the databases, ESTScan software [11] was used to deduce its sequence direction. GO and KEGG Orthology annotations of the unigenes were determined using Blast2Go, a universal tool for annotation, visualization, and analysis in functional genomics research [12], and InterProScan software.

### Differential gene expression (DGE) library preparation and sequencing

Equal amounts of RNA from three time points for developing ovules or abortive ovules were separately pooled for DGE profiling analysis. The total RNA extraction was carried out following the protocol previously mentioned. First, the two pooled total RNA samples were treated with DNase I to degrade any DNA from accidental contamination. Then the mRNA was enriched by using oligo (dT) magnetic beads. After it was mixed with fragmentation buffer, the mRNA was fragmented into short segments (about 200 bp). Then the first strand of cDNA was synthesized by using a random hexamer primer. Buffer, dNTPs, RNase H, and DNA polymerase I were added to synthesize the second strand. The double-stranded cDNA was purified with magnetic beads. End reparation was then performed and 3’-end single nucleotide A (adenine) was added. Finally, sequencing adaptors were ligated to the fragments. The fragments were enriched by PCR amplification. The Agilent 2100 Bioanalyzer and ABI StepOnePlus Real-Time PCR System were used to quantify and qualify the two sample libraries. The library products were then sequenced using the HiSeq 2000 Sequencing System (Illumina, Inc., USA). All raw data from the DGE library sequencing has been deposited in SRA (NCBI BioSample Accessions Number: SAMN03196408).

### Screening of DEGs

Genes that were differentially expressed among samples were screened, and GO functional enrichment analysis and KEGG pathway enrichment analysis were performed for these DEGs [13]. A strict algorithm identifying DEGs between two samples was developed to distinguish the significance of DGE. If the number of unambiguous clean tags from gene A is denoted x, given that every gene’s expression occupies only a small part of the library, p(x) will closely follow the Poisson distribution.

$$p(x)=\frac{e^{-\lambda }\lambda^{x}}{x!}\;\left(\lambda \text{ is the real transcript of the gene}\right)$$

The total clean tag number of the sample 1 is N1, and the total clean tag number of sample 2 is N2. Gene A holds x tags in sample1 and y tags in sample2. The probability that gene A will be expressed equally between two samples may be calculated using the following equation:

$$\begin{array}{c} 2\sum_{i=0}^{i=y}p(i|x)\\ \text{Or }2\times (1-\sum_{i=0}^{i=y}p(i|x))\;(\text{if}\;\sum_{i=0}^{i=y}p(i|x)>0.5)\\ p(y|x)={\left(\frac{N_{2}}{N_{1}}\right)}^{y}\frac{(x+y)!}{x!y!{\left(1+\frac{N_{2}}{N_{1}}\right)}^{\left(x+y+1\right)}} \end{array}$$

The *P*-value corresponds to the DGE test. False Discovery Rate (FDR) is a method to determine the threshold of the *P*-value in multiple tests [14]. FDR ≤ 0.001 and the absolute value of log_2_Ratio ≥ 1 were the thresholds to judge the significance of gene expression difference.

### Quantitative reverse transcription (qRT)-PCR validation

Total RNA from abortive and developing ovules was extracted and pooled as described for the DGE library previously mentioned, and three replicate experiments were prepared in each sample. First, reverse transcription to cDNA was performed on 100 ng total RNA using the PrimeScript RT reagent Kit (TaKaRa, Tokyo, Japan), following the manufacturer’s instructions. qRT-PCR was performed with 1μL cDNA template, 1× reaction buffer, 0.2 mM dNTP mixture, 0.4 μM each primer, 0.6× SYBR Green I dye (Generay Biotech), and 0.3 units of rTaqDNA polymerase (TaKaRa) in a Rotor-Gene 2000 thermocycler (Corbett Research, Sydney, Australia) using the SYBR Premix Ex Taq Kit (TaKaRa), according to the manufacturer’s protocol. Three technical replicates were run for each experiment. The cycle threshold (CT) values for each gene were normalized to the housekeeping gene β-actin using the formula (Unigene-ACTIN), where ACTIN is the mean CT of triplicate β-actin genes runs; Unigene is the mean CT of triplicate runs of the unigene of interest. Transcript fold-changes describing the change in expression of the target gene in samples from abortive ovules relative to that of the developing ovules transcript were calculated using the 2^−ΔΔCt^ method described by Schmittgen and Livak [15]. Results of qPCR were analyzed using one-way ANOVA followed by Dunett’s multiple comparison tests in SAS version 8.01 (SAS Institute, Inc., Cary, NC, USA). P values lower than 0.05 were considered significant. The error bars indicate the standard deviation from three different experiments. The primers used in qRT-PCR analysis are listed in S1 Table.

## Results

### Illumina sequencing and sequence assembly

**Fig 1:**
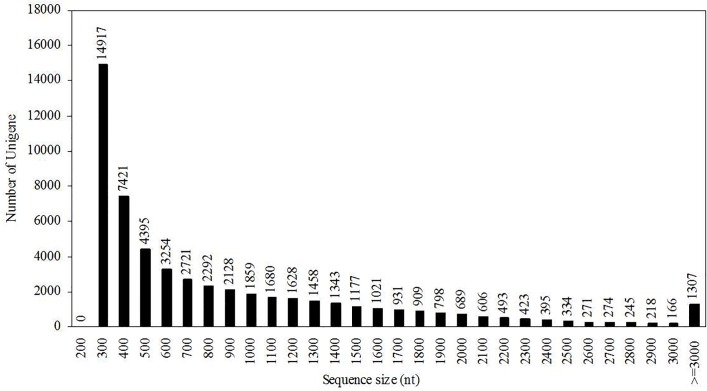
Unigene size distribution. All of the unigene sizes were calculated.

A total of 54,000,000 raw reads were generated after Illumina sequencing analysis. Reads with adaptors, unknown nucleotides larger than 5%, and low-quality reads with more than 20% low-quality bases (base quality ≤ 10) were removed. A total of 48,585,250 clean reads with 4,372,672,500 nucleotides (nt) was obtained with a Q20 percentage of 98.17%. The final sequence assembly results using Trinity [9] was 55,353 unigenes, including 18,751 clusters and 36,602 singletons, with a mean length of 844 nt. The unigene size distribution is shown in Fig 1, which indicates that unigenes shorter than 2000 nt accounted for 91.45% of the total unigenes.

### Annotation of predicted proteins

**Fig 2:**
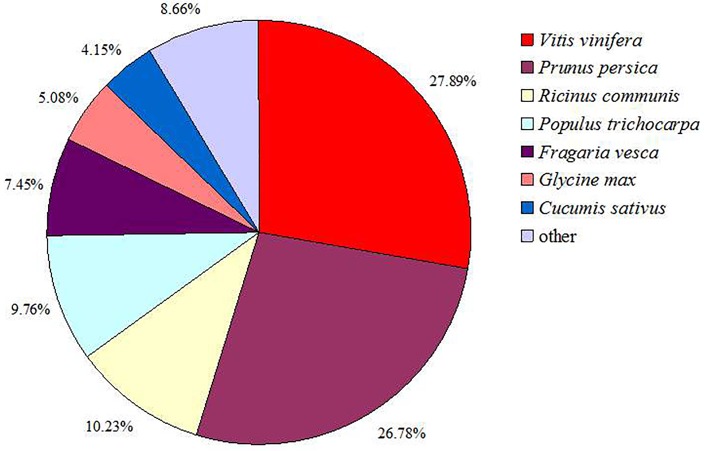
Species distribution of the BLASTX search results. This figure shows the species distribution of unigene BLASTX results against the NR protein database with a cutoff E-value < 10^−5^ and also shows the proportions of each species. Different colors represent different species. Species with proportions of less than 1% are not shown.

In order to annotate the unigenes, the unigene sequences were searched against the NR, NT, SwissProt, KEGG, COG, and GO databases with a cutoff E-value < 10^−5^. Annotation statistical data indicated that 37,080; 34,002; 23,585; 21,385; 13,345; and 28,642 unigenes were annotated, respectively. The 37,080 unigenes annotation results searched against the NR database are listed in S2 Table. In total, 38,810 unigenes returned a BLASTX result with a cutoff E-value < 10^−5^. The species distribution of the best match results were translated from BLASTX results as indicated in Fig 2. The *C*. *heterophylla* Fisch sequences showed the closest matches with *Vitis vinifera* (27.89%), followed by *Prunus persica* (26.78%), *Ricinus communis* (10.23%), *Populus trichocarpa* (9.76%), *Fragaria vesca* subsp. *vesca* (7.45%), and *Glycine max* (4.15%).

### GO and KEGG ontology classification

**Fig 3:**
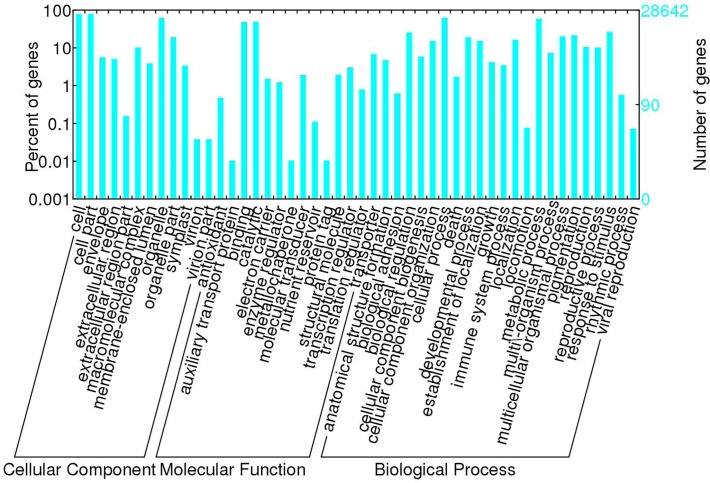
Gene Ontology (GO) categories of unigenes. Unigenes were annotated in three categories: cellular components, molecular functions, and biological processes.

The Blast2GO program [12] was used to obtain GO annotation of the unigenes on the basis of the NR annotation data. Unigenes with GO annotation accounted for 51.74% of all the unigenes and are listed in S3 Table. The GO categories of the unigenes are shown in Fig 3. The terms “cell” and “cell part,” “binding” and “catalytic,” and “process” and “locomotion” were dominant in the categories of cellular components, molecular functions, and biological processes, respectively. All unigenes were queried against the KEGG pathway database, and 38.63% of the 55,353 unigenes were given pathway annotations that were related to 128 pathways, including metabolism, plant hormone signal transduction, RNA transport, and purine metabolism. Unigenes with KEGG annotation are listed in S4 Table.

### Sequencing of two DGE libraries

The empty library and full cDNA library that were generated from the developing and abortive ovules, respectively, of *C*. *heterophylla* Fisch were sequenced and generated approximately 7.4 million raw reads each. Dirty reads, which contained adapters, unknown bases, or low-quality bases, and which constituted fewer than 1% of the total reads, were filtered out. Next, clean reads of each library were mapped to the assembled unigenes on the basis of the results of transcriptome analysis. The number of total mapped reads was about 6.4 to 6.5 million base pairs, accounting for approximately 87% to 88% of total reads (Table 1). The percentage of total unmapped reads was about 12% in the two libraries.

**Table 1:** Summary of mapping results.

Sample ID	Total reads^1^	Total base pairs^2^	Total mapped reads^3^	Perfect match^4^	< = 2 bp Mismatch^5^	Unique match^6^	Multi-position match^7^	Total unmapped reads^8^
Empty^9^	7,456,236	365,355,564	6,565,172	5,323,902	1,241,270	5,187,188	1,377,984	891,064
	-100%	-100%	-88.05%	-71.40%	-16.65%	-69.57%	-18.48	-11.95%
Full^10^	7,422,728	363,713,672	6,461,102	5,029,053	1,432,049	5,021,287	1,439,815	961,626
	-100%	-100%	-87.04%	-67.75%	-19.29%	-67.75%	-19.4	-12.96%

^1^ Total Reads: all reads included in this study.

^2^ Total BasePair: all nucleotides in this study.

^3^ Total Mapped Reads: number of reads that are similar in sequence to part of reference.

^4^ Perfect Match: portion of total mapped reads that can be perfectly mapped to reference.

^5^ < = 2 bp Mismatch: portion of total mapped reads that can be mapped to reference with < = 2 bp mismatches.

^6^ Unique Match: portion of total mapped reads that have only one mapped site in reference.

^7^ Multi-position Match: portion of total mapped reads that have multiple mapped sites in reference.

^8^ Total Mapped Reads: number of reads that have no similar sequences as any part of reference.

^9^ Empty: cDNA library generated from abortive ovules.

^10^ Full: cDNA library generated from developing ovules.

**Fig 4:**
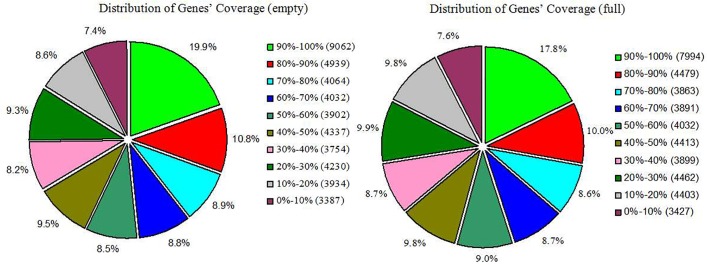
Genes’ coverage distribution of empty and full ovules in hazelnut. The numbers preceding the parentheses indicate the percentage of unique reads in each category, and the data in parentheses indicate the number of unique reads.

In total, 45,641 unigenes in the empty library and 44,863 unigenes in the full library were mapped to our transcriptome reference database. The average length of mapped unigenes was about 800 bp, and their average coverage was about 55% (Table 2). The gene expression level was calculated using the RPKM method [16]. The RPKM method eliminates the influence of different gene lengths and sequencing discrepancies on the calculation of gene expression level. The calculated values of the gene expression level may be directly used to compare the differences in gene expression among samples. The reads per kb per million reads of mapped unigenes was about 19 (Table 2). Detailed information about unigenes in the empty and full libraries that could be mapped to our transcriptome reference database is listed in S5 and S6 Tables, respectively. Coverage represents the percentage of a gene covered by reads, and the genes’ distribution coverage of the empty and full libraries is shown in Fig 4.

**Table 2:** Summary of unigenes mapped to transcriptome reference database.

Sample	NUMT^1^	Average length^2^ (bp)	Average coverage^3^ (%)	Average RPKM^4^
Empty^5^	45,641	797.68	56.52	19.38
Full^6^	44,863	802.76	54.47	19.69

^1^ NUMT: Number of unigenes mapped to transcriptome reference database.

^2^ Average length: average length of gene.

^3^ Average coverage: average coverage of gene.

^4^ Average RPKM: average reads per kb per million reads.

^5^ Empty: cDNA library generated from empty ovules.

^6^ Full: cDNA library generated from developing ovules.

### Variations in gene expression between developing and abortive ovules

**Fig 5:**
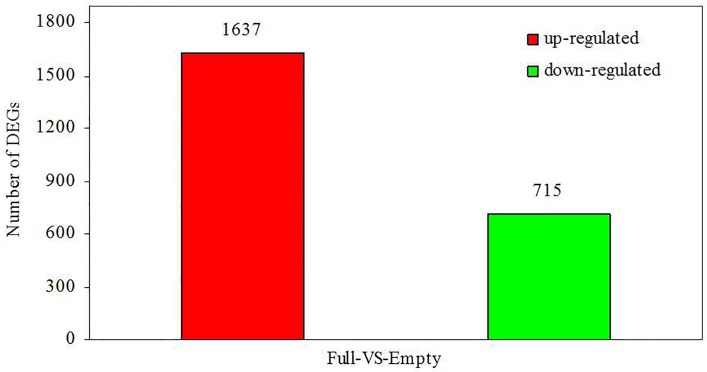
Overview of differential expression. Upregulated (red) and downregulated (green) unigenes were quantified.

In order to determine the variations in gene expression between developing and abortive ovules, the empty and full libraries were compared. FDR ≤ 0.001 and the absolute value of log_2_Ratio ≥ 1 were used as the threshold to judge the significance of gene expression difference. Comparative analysis revealed 2,352 significantly differentially expressed unigenes between the empty and full libraries. Among these unigenes, 1,637 were upregulated and 715 were downregulated in the empty library, compared with the full library (Fig 5). The significantly differentially expressed unigenes (S7 Table) were further analyzed to determine their biological and molecular functions. Their functional categories were assembled according to information in several public databases and related literature.

In the GO ontology, the terms “cell” and “cell part” were dominant in the category of cellular components, the term “catalytic activity” was dominant in the category of molecular functions, and the terms “cellular process” and “metabolic process” were dominant in the category of biological processes (S8 Table). According to GO classification, many of the differentially expressed genes were associated with oxidoreductase, peroxidase, and antioxidant activity (S9 Table).

Among 2,352 DEGs, 1,209 DEGs with pathway annotation were identified and classified into 116 distinct categories (S10 Table). The top five pathways were metabolic pathways (33.66%), biosynthesis of secondary metabolites pathways (18.86%), plant–pathogen interaction pathways (10.84%), plant hormone signal transduction pathways (7.69%), and phenylpropanoid biosynthesis pathways (5.46%). DEGs were involved in all of the plant hormone signal transduction pathways. DEGs in both libraries often were involved in the same hormone signal transduction pathways, including those of auxin, cytokine, gibberellin, ethylene, and jasmonic acid. However, all the DEGs in the empty library that were involved in the abscisic acid and salicylic acid signal pathways were upregulated. In the abscisic acid signal pathway, these changes included abscisic acid receptor (*PYL*) (Unigene4733_D2), protein phosphatase 2C (*PP2C*) (CL2293.Contig2_D2, CL3319.Contig2_D2, CL7150.Contig2_D2, and CL6767.Contig1_D2)), and ABA responsive element binding factor (*ABF*) (Unigene16189_D2). In the ethylene regulating signal pathway, these upregulation changes included serine/threonine-protein kinase CTR1 (*CTR1*) (Unigene19967_D2, CL102.Contig7_D2, Unigene16685_D2), ethylene-insensitive protein 3 (*EIN3*), ethylene-responsive transcription factor 1 (*ERF1*) (Unigene31203_D2, Unigene15326_D2) and ethylene-responsive transcription factor 2 (*ERF2*) (Unigene15326_D2, CL3888.Contig2_D2). In this study, we focused on a group of downregulated DEGs involved in embryo development (Table 3). According to GO classification and KEGG annotation, these DEGs execute important biological functions, including the generation of late embryogenesis abundant protein (*Catharanthus roseus*) (*LEA*) (CL97.Contig2_D2), actin-like protein 6B (*ACTL6B*) (CL5483.Contig2_D2), transformer-2 protein (TRA2) (CL97.Contig1_D2), transcription elongation factor SPT5 (*SUPT5H*) (Unigene22699_D2), histone deacetylase 1/2 (*HDAC1_2*) (CL2945.Contig1_D2), brassinosteroid insensitive 1-associated receptor kinase 1 (*BAK1*) (CL3163.Contig3_D2), amidophosphoribosyltransferase (*purF*) (Unigene17483_D2), protein-serine/threonine kinase (*E2*.*7*.*11*.*-*) (Unigene18327_D2), transport inhibitor response 1 (*TIR1*) (Unigene19803_D2), superoxide dismutase, Fe–Mn family (*SOD2*) (Unigene18238_D2), polypeptide N-acetylglucosaminyltransferase (*OGT*) (CL3630.Contig1_D2), and DNA polymerase epsilon subunit 3 (*POLE3*) (Unigene18285_D2). Path annotation indicated that these DEGs were included in several different pathways.

**Table 3:** Downregulated differentially expressed genes that are probably involved in embryo development.

Gene ID	Gene length^1^	Full RPKM^2^	Empty RPKM^3^	log_2_ Ratio^4^	Up-Down^5^	P-value^6^	FDR^7^	Annotation
CL97.Contig2_D2	727	52	1	-5.75	Down	2.60E-15	1.06E-13	Late embryogenesis abundant protein
CL97.Contig1_D2	760	21	1	-4.44	Down	4.05E-06	6.10E-05	Late embryogenesis abundant protein [*Catharanthus roseus*]
Unigene22699_D2	933	298	38	-3.02	Down	2.75E-53	4.28E-51	Transformer-2 protein (*TRA2*)
CL2945.Contig1_D2	450	30	4	-2.95	Down	2.21E-06	3.51E-05	Transcription elongation factor SPT5 (*SUPT5H*)
CL3163.Contig3_D2	1041	161	28	-2.57	Down	4.35E-25	3.02E-23	Histone deacetylase 1/2 (*HDAC1_2*)
Unigene17483_D2	1258	69	24	-1.57	Down	1.01E-06	1.70E-05	Brassinosteroid insensitive 1-associated receptor kinase 1 (*BAK1*)
Unigene18327_D2	629	68	25	-1.49	Down	3.01E-06	4.64E-05	Amidophosphoribosyltransferase (*purF*)
Unigene19803_D2	1748	131	53	-1.35	Down	1.53E-09	3.82E-08	Protein-serine/threonine kinase transport inhibitor response 1 (*TIR1*)
Unigene18238_D2	953	1282	537	-1.3	Down	1.18E-75	2.62E-73	Superoxide dismutase, Fe-Mn family (*SOD2*)
CL3630.Contig1_D2	1710	237	111	-1.14	Down	1.08E-12	3.60E-11	polypeptide N-Acetylglucosaminyltransferase (*OGT*)
Unigene18285_D2	951	890	435	-1.08	Down	7.83E-40	9.01E-38	DNA polymerase epsilon subunit 3 (*POLE3*)

^1^ Gene length: length of all exons in gene

^2^ Full RPKM: reads per kb per million reads of cDNA library generated from developing ovules

^3^ Empty RPKM: reads per kb per million reads of cDNA library generated from abortive ovules

^4^ log_2_ Ratio (empty RPKM / full RPKM), log_2_ (Fold Change)

^5^ Up-/down-regulation (empty/full): empty is up-/down-regulation in empty library relative to full library

^6^ P-value: p-value for hypothesis testing

^7^ FDR: false discovery rate.

### Confirmation through qRT-PCR

**Fig 6:**
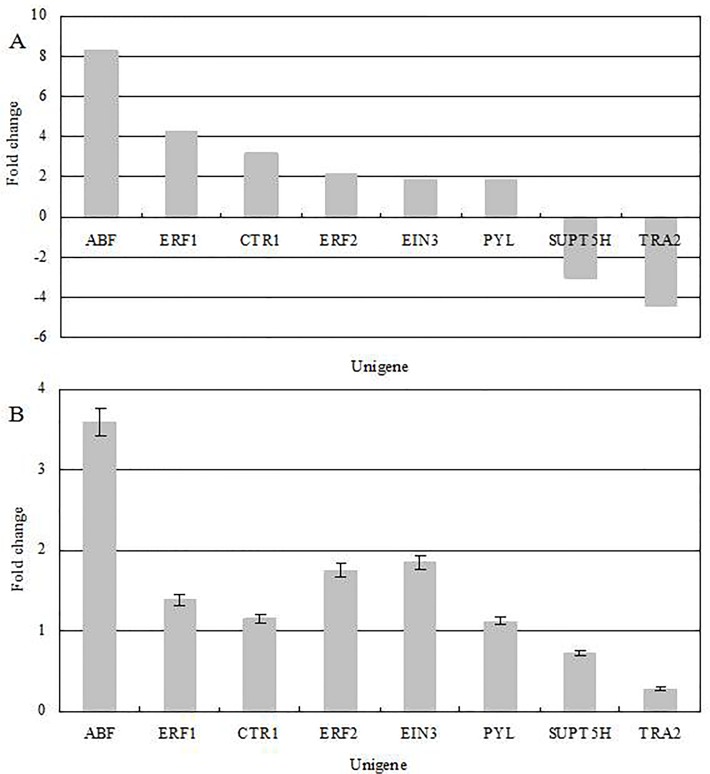
(A) Gene expression data for digital gene expression analysis. The fold changes of the genes were calculated as the log2 value of each empty/full comparison and are shown on the y-axis. (B) The quantitative real-time PCR analysis of gene expression data. Quantitative variation of *ABF*, *ERF1*, *CTR1*, *ERF2*, *EIN3*, *PYL*, *SUPT5H* and *TRA2* in Empty were compared with Full, and β-Actin (GenBank: AB298788) was used as the internal standard.

To evaluate the validity of Illumina analysis, six upregulated DEGs involved in abscisic acid and ethylene signal pathways, including *ABF* (ABA-responsive element-binding factor), *ERF1* (Ethylene-responsive transcription factor 1), *CTR1* (serine/threonine-protein kinase CTR1), *ERF2* (Ethylene-responsive transcription factor 2) *EIN3* (Ethylene-insensitive protein 3) and *PYL* (Abscisic acid receptor PYR/PYL family), two downregulated DEGs which involved in embryo development, including *TRA2* (transformer-2 protein) and *SUPT5H* (transcription elongation factor SPT5), were selected for examination by real-time RT-PCR. All the genes mentioned are probably involved in the dormancy and senescence of seed or embryo development. Information about these genes and their gene-specific primers is shown in S1 Table. The DGE results (Fig 6A) for these genes were identical to those obtained by RT-PCR (Fig 6B).

## Discussion

Our analyses showed that *C*. *heterophylla* Fisch shared the highest similarity with grape (*V*. *vinifera*), peach (*P*. *persica*), castor bean (*R*. *communis*), poplar (*P*. *trichocarpa*), strawberry (*F*. *vesca* subsp. *vesca*), and soybean (*G*. *max*) in the BLAST annotation. High similarity of proteins in the European hazelnut variety ‘Jefferson’ to sequences in related plants demonstrated that this variety shared 80.8% similarity with grape (*Vinifera* L.), poplar (*P*. *trichocarpa* Torr. & A. Gray), and castor bean (*R*. *communis* L.) sequences [17]. *C*. *mandshurica* sequences shared 34% similarity with *V*. *vinifera*, followed by *P*. *trichocarpa* (18%) and *R*. *communis* (17%) [18]. According to our results, species of the genus *Corylus* show the most similarity with grape (*Vinifera* L.), poplar (*P*. *trichocarpa*), and castor bean (*R*. *communis*).

In order to better understand the gene expression information in ovules of *C*. *heterophylla* Fisch, two cDNA libraries generated from developing ovules and abortive ovules were constructed during the ovule development stage to acquire complete transcriptome information. The empty and full DEG libraries were created to reveal the DEGs related to embryo development. We obtained a total of 55,353 unigenes. The mean length of unigenes was 844 nt and the N50 was 1,347 bp, which was much better than the assembled sequence quality of the EST database (28,255 contigs with an average length of 532 bp and an N50 of 961) [17]. Furthermore, our unigene data assembled from transcriptome sequencing data have far fewer redundant sequences than do the EST data. In addition, RNA samples used in the present study were extracted from the ovules of nuts rather than the leaves of young seedlings, which enrich the current *Corylus* database and aids future studies of the function of genes involved in hazelnut fruit development.

The DEGs were involved in all of the plant hormone signal transduction pathways. In the present study, we specifically focused on the abscisic acid and ethylene signal pathways (Table 4), which were probably involved in the dormancy and senescence of seed. In the abscisic acid signal pathway, all three DEGs were upregulated, including abscisic acid receptor (*PYL*), protein phosphatase 2C (*PP2C*), and ABA-responsive element binding factor (*ABF*). PYLs are ABA receptors functioning at the apex of a negative regulatory pathway that controls ABA signaling by inhibiting type 2C PP2Cs [19]. The upregulation of *ABF* is believed to enhance abiotic stress signaling in rice [20]. The upregulation of *PYL*, *PP2C*, and *ABF* likely induce embryo dormancy through the ABA signal pathway. In addition, four DEGs were involved in the ethylene-regulating signal pathway, including serine/threonine-protein kinase CTR1 (*CTR1*), ethylene-insensitive protein 3 (*EIN3*), ethylene-responsive transcription factor 1 (*ERF1*), and ethylene-responsive transcription factor 2 (*ERF2*). Among these, *EIN3* is thought to be involved in ubiquitin-mediated proteolysis, and the nuclear proteins ERF1/2 act on downstream components of *EIN3* in the ethylene signaling pathway. All four proteins acted sequentially in a cascade of transcriptional regulation initiated by ethylene gas [21]. Although the ovules used in the study were far from mature, the activation of the ethylene signaling pathway is likely to result in the senescence of the young ovule and embryo.

**Table 4:** The DEGs involved in abscisic acid and ethylene signal pathways.

Gene ID	Gene length^1^	Full RPKM^2^	Empty RPKM^3^	log_2_Ratio^4^	Up-Down^5^	P-value^6^	FDR^7^	Annotation
Unigene16189_D2	854	0.01	3.16	8.30	Up	7.77E-05	8.93E-04	ABA-responsive element binding factor
Unigene4733_D2	380	5.76	20.29	1.82	Up	5.87E-05	6.96E-04	Abscisic acid receptor PYR/PYL family
CL7150.Contig2_D2	1572	4.56	14.35	1.65	Up	8.33E-11	2.35E-09	Protein phosphatase 2C
Unigene31203_D2	388	0.51	9.94	4.28	Up	1.52E-05	2.06E-04	Ethylene-responsive transcription factor 1
Unigene15326_D2	573	0.70	9.76	3.81	Up	3.87E-07	6.97E-06	Ethylene-responsive transcription factor 1 or 2
Unigene19967_D2	991	1.00	9.34	3.22	Up	7.93E-10	2.03E-08	Serine/threonine-protein kinase CTR1
Unigene26530_D2	2242	19.99	72.83	1.87	Up	3.62E-81	8.69E-79	Ethylene-insensitive protein 3

^1^ Gene length: length of all exons in gene.

^2^ Full RPKM: reads per kb per million reads of cDNA library generated from developing ovules.

^3^ Empty RPKM: reads per kb per million reads of cDNA library generated from abortive ovules.

^4^ log_2_Ratio (empty RPKM /full RPKM), log_2_ (Fold Change).

^5^ Up-/downregulation (empty/full): empty is up-/downregulation in empty library relative to full library.

^6^ P-value: p-value for hypothesis testing.

^7^ FDR: false discovery rate.

In addition to a cascade of DEGs in the plant hormone signal pathway, we also identified some important downregulated DEGs that were involved in the regulation of embryo development according to GO enrichment analysis. First, two important protective proteins in the abortive ovule were downregulated. Late embryogenesis abundant protein (*LEA*) in plants protects other proteins from aggregation due to desiccation or osmotic stresses associated with low temperature; in particular, it protects mitochondrial membranes against dehydration damage [22–23]. Superoxide dismutase (*SOD2*) is a protein cofactored with either iron or manganese, which catalyzes the dismutation of superoxide (O_2_^−^) into oxygen and hydrogen peroxide. It is an important antioxidant enzyme that functions in the defense of nearly all cells exposed to oxygen. Thus, in the abortive ovule, these protective proteins fail to execute their defensive functions, which reduce the survival or development of the embryo.

Second, some important proteins involved in the splicing of mRNA, RNA transcription, DNA synthesis, and protein modification were significantly downregulated. Among them, transformer-2 protein (*TRA2*) is an essential component of a splicing enhancer complex, and transcription elongation factor SPT5 (*SUPT5H*) and DNA polymerase epsilon subunit 3 (*POLE3*) are important components involved in RNA transcription and DNA duplication, respectively, while amidophosphoribosyltransferase (*purF*) catalyzes the first step of de novo purine nucleotide biosynthesis. Brassinosteroid (BR) insensitive 1-associated receptor kinase 1 (*BAK1*) positively regulates the BR-dependent plant growth pathway and negatively regulates the BR-independent cell-death pathway, and its downregulation would activate the cell death pathway in young ovules and embryos of hazelnut. Finally, several DEGs coding for important components such as actin-like protein 6B (*ACTL6B*) and histone deacetylase 1/2 (*HDAC1_2*) were downregulated, and plants in which transport inhibitor response 1 (*TIR1*) is disrupted are deficient in a variety of auxin-regulated growth processes, many of which may be involved in the abortive process of ovules and embryos in hazelnut.

A large number of DEGs were not identified by KEGG annotation, GO annotation, or BLAST NR results. At the same time, the functions of many DEGs cannot be identified due to mismatched annotations in mammals or microorganisms. These may be identified at some point in the future as more annotations become available in public databases. This study is the first report of the involvement of DEGs in the embryo development pathway of *Corylus*, and our findings facilitate a better understanding of the developmental process in hazelnut, which may improve our ability to obtain high yields from this economically important crop.

## Conclusions

Two cDNA libraries generated from developing ovules and abortive ovules were constructed during the ovule development stage in order to acquire complete transcriptome information. Sequencing and assembly results revealed 55,353 unigenes, including 18,751 clusters and 36,602 singletons. Transcriptome analysis results allowed gene expression changes between developing and abortive ovules in hazelnut to be compared. The results of the comparison indicated that a total of 1,637 and 715 unigenes were significantly upregulated and downregulated in the empty library compared with the full library. Among 2,352 DEGs, 1,209 DEGs with pathway annotation were identified and classified into 116 distinct categories. The top five pathways were metabolic pathways (33.66%), biosynthesis of secondary metabolites pathways (18.86%), plant–pathogen interaction pathways (10.84%), plant hormone signal transduction pathways (7.69%), and phenylpropanoid biosynthesis pathways (5.46%). We particularly focused on two groups of genes that were probably involved in the dormancy and senescence of seed and embryo development. (1) *PYL*, *PP2C*, and *ABF* in the abscisic acid pathway and *CTR1*, *EIN3*, *ERF1*, and *ERF2* in the ethylene signal pathway were upregulated in the abortive ovule, and these changes may trigger the abscisic and ethylene signal pathways to induce the dormancy or senescence of ovules in hazelnut. (2) Some important proteins, including *LEA*, *SOD2*, *TRA2*, *SUPT5H*, *POLE3*, *purF*, *BAK1*, *ACTL6B*, and *HDAC1_2* was downregulated in the abortive ovule, which probably damaged the protective function, activated the cell death pathway, and blocked development in the abortive ovule.

## Supporting Information

S1 Table Primers used in quantitative real-time PCR for validation of differentially expressed genes. (XLS)

S2 Table Top hits obtained by BLASTX for the unigenes. (XLS)

S3 Table Unigenes with Gene Ontology (GO) annotation. (XLS)

S4 Table Unigenes with Kyoto Encyclopedia of Genes and Genomes (KEGG) annotation. (XLS)

S5 Table Unigenes of empty library that could be mapped to our transcriptome reference database. (XLS)

S6 Table Unigenes of full library that could be mapped to our transcriptome reference database. (XLS)

S7 Table Significantly differentially expressed unigenes. (XLS)

S8 Table Gene Ontology (GO) categories of the differentially expressed genes. (SVG)

S9 Table Gene Ontology (GO) term enrichment analysis of the differentially expressed genes. (DOC)

S10 Table Kyoto Encyclopedia of Genes and Genomes (KEGG) pathway enrichment analysis of differentially expressed genes. (HTM)
