# Supplementary material for: Transcriptome Analysis and Gene Expression Profiling of Abortive and Developing Ovules during Fruit Development in Hazelnut
Source: PLoS One. 2015 Apr 2;10(4):e0122072. doi: 10.1371/journal.pone.0122072 (PMC4383543; doi:10.1371/journal.pone.0122072)
Supplement: S9 Table — (DOC) [file pone.0122072.s009.doc]

**Table S9** GO term enrichment analysis of the differentially expressed genes

|  | **Gene Ontology term** | **Cluster frequency** | **Corrected P-value** |
| --- | --- | --- | --- |
| Cellular Component | external encapsulating structure | 101 out of 1018 genes, 9.9% | 4.77e-19 |
|  | [cell wall](http://amigo.geneontology.org/cgi-bin/amigo/go.cgi?action=query&view=query&query=GO:0005618&search_constraint=terms) | 96 out of 1018 genes, 9.4% | 1.13e-17 |
|  | [extracellular region](http://amigo.geneontology.org/cgi-bin/amigo/go.cgi?action=query&view=query&query=GO:0005576&search_constraint=terms) | 142 out of 1018 genes, 13.9% | 2.21e-17 |
|  | cell periphery | 339 out of 1018 genes, 33.3% | 7.47e-16 |
|  | [vacuole](http://amigo.geneontology.org/cgi-bin/amigo/go.cgi?action=query&view=query&query=GO:0005773&search_constraint=terms) | 113 out of 1018 genes, 11.1% | 1.54e-09 |
|  | [membrane](http://amigo.geneontology.org/cgi-bin/amigo/go.cgi?action=query&view=query&query=GO:0016020&search_constraint=terms) | 469 out of 1018 genes, 46.1% | 2.06e-08 |
|  | [plasma membrane](http://amigo.geneontology.org/cgi-bin/amigo/go.cgi?action=query&view=query&query=GO:0005886&search_constraint=terms) | 279 out of 1018 genes, 27.4% | 9.02e-08 |
|  | [apoplast](http://amigo.geneontology.org/cgi-bin/amigo/go.cgi?action=query&view=query&query=GO:0048046&search_constraint=terms) | 52 out of 1018 genes, 5.1% | 1.78e-07 |
|  | [intrinsic to membrane](http://amigo.geneontology.org/cgi-bin/amigo/go.cgi?action=query&view=query&query=GO:0031224&search_constraint=terms) | 178 out of 1018 genes, 17.5% | 4.39e-06 |
|  | plant-type cell wall | 36 out of 1018 genes, 3.5% | 2.29e-05 |
|  | chorion | 5 out of 1018 genes, 0.5% | 0.00017 |
|  | [micropyle](http://amigo.geneontology.org/cgi-bin/amigo/go.cgi?action=query&view=query&query=GO:0070825&search_constraint=terms) | 5 out of 1018 genes, 0.5% | 0.00017 |
|  | [integral to membrane](http://amigo.geneontology.org/cgi-bin/amigo/go.cgi?action=query&view=query&query=GO:0016021&search_constraint=terms) | 159 out of 1018 genes, 15.6% | 0.00029 |
|  | [membrane part](http://amigo.geneontology.org/cgi-bin/amigo/go.cgi?action=query&view=query&query=GO:0044425&search_constraint=terms) | 198 out of 1018 genes, 19.4% | 0.00081 |
|  | [lipid particle](http://amigo.geneontology.org/cgi-bin/amigo/go.cgi?action=query&view=query&query=GO:0005811&search_constraint=terms) | 5 out of 1018 genes, 0.5% | 0.00622 |
| Molecular Function | [oxidoreductase activity](http://amigo.geneontology.org/cgi-bin/amigo/go.cgi?action=query&view=query&query=GO:0016491&search_constraint=terms) | 257 out of 1161 genes, 22.1% | 3.06e-14 |
|  | [peroxidase activity](http://amigo.geneontology.org/cgi-bin/amigo/go.cgi?action=query&view=query&query=GO:0004601&search_constraint=terms) | 28 out of 1161 genes, 2.4% | 1.88e-08 |
|  | [oxidoreductase activity, acting on peroxide as acceptor](http://amigo.geneontology.org/cgi-bin/amigo/go.cgi?action=query&view=query&query=GO:0016684&search_constraint=terms) | 28 out of 1161 genes, 2.4% | 1.88e-08 |
|  | [antioxidant activity](http://amigo.geneontology.org/cgi-bin/amigo/go.cgi?action=query&view=query&query=GO:0016209&search_constraint=terms) | 31 out of 1161 genes, 2.7% | 1.58e-07 |
|  | [iron ion binding](http://amigo.geneontology.org/cgi-bin/amigo/go.cgi?action=query&view=query&query=GO:0005506&search_constraint=terms) | 59 out of 1161 genes, 5.1% | 3.82e-06 |
|  | [heme binding](http://amigo.geneontology.org/cgi-bin/amigo/go.cgi?action=query&view=query&query=GO:0020037&search_constraint=terms) | 43 out of 1161 genes, 3.7% | 3.87e-05 |
|  | [hydrolase activity, acting on glycosyl bonds](http://amigo.geneontology.org/cgi-bin/amigo/go.cgi?action=query&view=query&query=GO:0016798&search_constraint=terms) | 62 out of 1161 genes, 5.3% | 4.52e-05 |
|  | [serine-type carboxypeptidase activity](http://amigo.geneontology.org/cgi-bin/amigo/go.cgi?action=query&view=query&query=GO:0004185&search_constraint=terms) | 14 out of 1161 genes, 1.2% | 5.74e-05 |
|  | [hydrolase activity, hydrolyzing O-glycosyl compounds](http://amigo.geneontology.org/cgi-bin/amigo/go.cgi?action=query&view=query&query=GO:0004553&search_constraint=terms) | 57 out of 1161 genes, 4.9% | 0.00013 |
|  | chitin binding | 7 out of 1161 genes, 0.6% | 0.00019 |
|  | tetrapyrrole binding | 43 out of 1161 genes, 3.7% | 0.00035 |
|  | serine-type exopeptidase activity | 14 out of 1161 genes, 1.2% | 0.00121 |
|  | oxidoreductase activity, acting on paired donors, with incorporation or reduction of molecular oxygen | 50 out of 1161 genes, 4.3% | 0.00141 |
|  | UDP-glucosyltransferase activity | 38 out of 1161 genes, 3.3% | 0.00172 |
|  | 2-alkenal reductase [NAD(P)] activity | 59 out of 1161 genes, 5.1% | 0.00270 |
|  | glucosyltransferase activity | 40 out of 1161 genes, 3.4% | 0.00338 |
|  | chitinase activity | 9 out of 1161 genes, 0.8% | 0.00394 |
|  | hydroperoxide dehydratase activity | 4 out of 1161 genes, 0.3% | 0.00452 |
|  | dioxygenase activity | 29 out of 1161 genes, 2.5% | 0.00586 |
|  | trehalose-phosphatase activity | 7 out of 1161 genes, 0.6% | 0.00851 |
|  | transmembrane transporter activity | 125 out of 1161 genes, 10.8% | 0.00899 |
| Biological Process | [oxidation-reduction process](http://amigo.geneontology.org/cgi-bin/amigo/go.cgi?action=query&view=query&query=GO:0055114&search_constraint=terms) | 237 out of 1157 genes, 20.5% | 1.43e-08 |
|  | [phenylpropanoid metabolic process](http://amigo.geneontology.org/cgi-bin/amigo/go.cgi?action=query&view=query&query=GO:0009698&search_constraint=terms) | 46 out of 1157 genes, 4.0% | 8.63e-08 |
|  | [response to chemical stimulus](http://amigo.geneontology.org/cgi-bin/amigo/go.cgi?action=query&view=query&query=GO:0042221&search_constraint=terms) | 311 out of 1157 genes, 26.9% | 3.35e-06 |
|  | [response to biotic stimulus](http://amigo.geneontology.org/cgi-bin/amigo/go.cgi?action=query&view=query&query=GO:0009607&search_constraint=terms) | 141 out of 1157 genes, 12.2% | 0.00012 |
|  | [secondary metabolite biosynthetic process](http://amigo.geneontology.org/cgi-bin/amigo/go.cgi?action=query&view=query&query=GO:0044550&search_constraint=terms) | 47 out of 1157 genes, 4.1% | 0.00016 |
|  | [secondary metabolic process](http://amigo.geneontology.org/cgi-bin/amigo/go.cgi?action=query&view=query&query=GO:0019748&search_constraint=terms) | 69 out of 1157 genes, 6.0% | 0.00022 |
|  | [response to other organism](http://amigo.geneontology.org/cgi-bin/amigo/go.cgi?action=query&view=query&query=GO:0051707&search_constraint=terms) | 136 out of 1157 genes, 11.8% | 0.00049 |
|  | [response to hormone stimulus](http://amigo.geneontology.org/cgi-bin/amigo/go.cgi?action=query&view=query&query=GO:0009725&search_constraint=terms) | 133 out of 1157 genes, 11.5% | 0.00068 |
|  | [response to endogenous stimulus](http://amigo.geneontology.org/cgi-bin/amigo/go.cgi?action=query&view=query&query=GO:0009719&search_constraint=terms) | 133 out of 1157 genes, 11.5% | 0.00092 |
|  | [phenylpropanoid biosynthetic process](http://amigo.geneontology.org/cgi-bin/amigo/go.cgi?action=query&view=query&query=GO:0009699&search_constraint=terms) | 31 out of 1157 genes, 2.7% | 0.00095 |
|  | [lignin metabolic process](http://amigo.geneontology.org/cgi-bin/amigo/go.cgi?action=query&view=query&query=GO:0009808&search_constraint=terms) | 17 out of 1157 genes, 1.5% | 0.00119 |
|  | [response to organic substance](http://amigo.geneontology.org/cgi-bin/amigo/go.cgi?action=query&view=query&query=GO:0010033&search_constraint=terms) | 204 out of 1157 genes, 17.6% | 0.00255 |
|  | [water transport](http://amigo.geneontology.org/cgi-bin/amigo/go.cgi?action=query&view=query&query=GO:0006833&search_constraint=terms) | 21 out of 1157 genes, 1.8% | 0.00460 |
|  | [fluid transport](http://amigo.geneontology.org/cgi-bin/amigo/go.cgi?action=query&view=query&query=GO:0042044&search_constraint=terms) | 21 out of 1157 genes, 1.8% | 0.00460 |
|  | [response to wounding](http://amigo.geneontology.org/cgi-bin/amigo/go.cgi?action=query&view=query&query=GO:0009611&search_constraint=terms) | 38 out of 1157 genes, 3.3% | 0.00766 |
