# Supplementary material for: Transcriptome Analysis and Gene Expression Profiling of Abortive and Developing Ovules during Fruit Development in Hazelnut
Source: PLoS One. 2015 Apr 2;10(4):e0122072. doi: 10.1371/journal.pone.0122072 (PMC4383543; doi:10.1371/journal.pone.0122072)
Supplement: S10 Table — (HTM) [file pone.0122072.s010.htm]

full-VS-empty

1. full-VS-empty

| # | Pathway | Sample1 (1209) | Sample2 (21385) | Pvalue | Qvalue | Pathway ID |
| 1 | Biosynthesis of secondary metabolites | 228 | 2179 | 5.391918e-21 | 6.254625e-19 | ko01110 |
| 2 | Metabolic pathways | 407 | 4838 | 1.039592e-19 | 6.029634e-18 | ko01100 |
| 3 | Phenylpropanoid biosynthesis | 66 | 352 | 4.458671e-18 | 1.724019e-16 | ko00940 |
| 4 | Phenylalanine metabolism | 35 | 126 | 1.535282e-15 | 4.452318e-14 | ko00360 |
| 5 | Plant-pathogen interaction | 131 | 1181 | 5.400729e-14 | 1.252969e-12 | ko04626 |
| 6 | Flavone and flavonol biosynthesis | 30 | 123 | 6.349745e-12 | 1.227617e-10 | ko00944 |
| 7 | Flavonoid biosynthesis | 36 | 191 | 1.535849e-10 | 2.545121e-09 | ko00941 |
| 8 | alpha-Linolenic acid metabolism | 28 | 134 | 1.479864e-09 | 2.145803e-08 | ko00592 |
| 9 | Stilbenoid, diarylheptanoid and gingerol biosynthesis | 25 | 155 | 2.011844e-06 | 2.593043e-05 | ko00945 |
| 10 | Alanine, aspartate and glutamate metabolism | 18 | 91 | 2.833662e-06 | 3.205390e-05 | ko00250 |
| 11 | Plant hormone signal transduction | 93 | 1018 | 3.039594e-06 | 3.205390e-05 | ko04075 |
| 12 | Endocytosis | 74 | 767 | 4.840422e-06 | 4.679075e-05 | ko04144 |
| 13 | Ether lipid metabolism | 59 | 579 | 8.812344e-06 | 7.863322e-05 | ko00565 |
| 14 | Glycerophospholipid metabolism | 73 | 770 | 1.029991e-05 | 8.534211e-05 | ko00564 |
| 15 | Zeatin biosynthesis | 23 | 152 | 1.504904e-05 | 1.163792e-04 | ko00908 |
| 16 | Carotenoid biosynthesis | 21 | 134 | 2.031360e-05 | 1.472736e-04 | ko00906 |
| 17 | Cyanoamino acid metabolism | 21 | 142 | 4.923955e-05 | 3.359875e-04 | ko00460 |
| 18 | Limonene and pinene degradation | 19 | 129 | 0.0001169055 | 7.533910e-04 | ko00903 |
| 19 | Fatty acid biosynthesis | 11 | 52 | 0.0001276101 | 7.790932e-04 | ko00061 |
| 20 | Other glycan degradation | 18 | 121 | 0.0001538427 | 8.922877e-04 | ko00511 |
| 21 | ABC transporters | 27 | 252 | 0.001100178 | 6.077174e-03 | ko02010 |
| 22 | Taurine and hypotaurine metabolism | 6 | 24 | 0.001798909 | 9.485157e-03 | ko00430 |
| 23 | Starch and sucrose metabolism | 48 | 552 | 0.002078368 | 1.041138e-02 | ko00500 |
| 24 | Benzoxazinoid biosynthesis | 8 | 42 | 0.002154078 | 1.041138e-02 | ko00402 |
| 25 | Pentose and glucuronate interconversions | 28 | 287 | 0.003572252 | 1.657525e-02 | ko00040 |
| 26 | Glyoxylate and dicarboxylate metabolism | 13 | 101 | 0.004501109 | 2.008187e-02 | ko00630 |
| 27 | Galactose metabolism | 15 | 130 | 0.006710801 | 2.883159e-02 | ko00052 |
| 28 | RNA polymerase | 39 | 462 | 0.008323501 | 3.448308e-02 | ko03020 |
| 29 | Diterpenoid biosynthesis | 8 | 53 | 0.009361027 | 3.744411e-02 | ko00904 |
| 30 | Glycosphingolipid biosynthesis - globo series | 3 | 9 | 0.01170664 | 4.526567e-02 | ko00603 |
| 31 | Indole alkaloid biosynthesis | 4 | 17 | 0.01338267 | 5.007709e-02 | ko00901 |
| 32 | Tyrosine metabolism | 12 | 104 | 0.01439157 | 5.094618e-02 | ko00350 |
| 33 | Cutin, suberine and wax biosynthesis | 10 | 80 | 0.01449331 | 5.094618e-02 | ko00073 |
| 34 | Biosynthesis of unsaturated fatty acids | 8 | 60 | 0.01913715 | 6.529145e-02 | ko01040 |
| 35 | Ascorbate and aldarate metabolism | 12 | 113 | 0.02602696 | 8.599979e-02 | ko00053 |
| 36 | Pyrimidine metabolism | 49 | 653 | 0.02668959 | 8.599979e-02 | ko00240 |
| 37 | Nitrogen metabolism | 9 | 77 | 0.02931695 | 9.191260e-02 | ko00910 |
| 38 | Glycolysis / Gluconeogenesis | 19 | 217 | 0.03918246 | 1.182165e-01 | ko00010 |
| 39 | Tropane, piperidine and pyridine alkaloid biosynthesis | 6 | 45 | 0.03991600 | 1.182165e-01 | ko00960 |
| 40 | Glycosphingolipid biosynthesis - ganglio series | 5 | 34 | 0.04076432 | 1.182165e-01 | ko00604 |
| 41 | Isoquinoline alkaloid biosynthesis | 5 | 36 | 0.05039761 | 1.425884e-01 | ko00950 |
| 42 | Glutathione metabolism | 12 | 126 | 0.05318665 | 1.468965e-01 | ko00480 |
| 43 | Betalain biosynthesis | 1 | 1 | 0.05653495 | 1.525129e-01 | ko00965 |
| 44 | Glucosinolate biosynthesis | 3 | 17 | 0.06775106 | 1.786164e-01 | ko00966 |
| 45 | Tryptophan metabolism | 6 | 54 | 0.0831313 | 2.142940e-01 | ko00380 |
| 46 | Isoflavonoid biosynthesis | 5 | 42 | 0.08659898 | 2.183800e-01 | ko00943 |
| 47 | Butanoate metabolism | 5 | 43 | 0.09367396 | 2.311953e-01 | ko00650 |
| 48 | Amino sugar and nucleotide sugar metabolism | 18 | 229 | 0.0990658 | 2.363345e-01 | ko00520 |
| 49 | Linoleic acid metabolism | 7 | 70 | 0.09983095 | 2.363345e-01 | ko00591 |
| 50 | Fatty acid metabolism | 8 | 86 | 0.1127430 | 2.615638e-01 | ko00071 |
| 51 | Pyruvate metabolism | 13 | 160 | 0.1203529 | 2.737439e-01 | ko00620 |
| 52 | Anthocyanin biosynthesis | 2 | 11 | 0.1252417 | 2.793853e-01 | ko00942 |
| 53 | Purine metabolism | 45 | 674 | 0.1398716 | 3.061341e-01 | ko00230 |
| 54 | Regulation of autophagy | 9 | 106 | 0.1453898 | 3.123188e-01 | ko04140 |
| 55 | Glycerolipid metabolism | 10 | 124 | 0.1639761 | 3.458405e-01 | ko00561 |
| 56 | Brassinosteroid biosynthesis | 3 | 26 | 0.1795687 | 3.719637e-01 | ko00905 |
| 57 | Glycosaminoglycan degradation | 5 | 54 | 0.1886518 | 3.839230e-01 | ko00531 |
| 58 | Monoterpenoid biosynthesis | 2 | 15 | 0.2067857 | 4.083367e-01 | ko00902 |
| 59 | Biotin metabolism | 1 | 4 | 0.2076885 | 4.083367e-01 | ko00780 |
| 60 | Cysteine and methionine metabolism | 11 | 150 | 0.228883 | 4.425071e-01 | ko00270 |
| 61 | Fructose and mannose metabolism | 9 | 123 | 0.2604486 | 4.952793e-01 | ko00051 |
| 62 | Vitamin B6 metabolism | 2 | 20 | 0.313524 | 5.865933e-01 | ko00750 |
| 63 | Other types of O-glycan biosynthesis | 3 | 37 | 0.3488198 | 6.422714e-01 | ko00514 |
| 64 | Ubiquinone and other terpenoid-quinone biosynthesis | 7 | 102 | 0.3549967 | 6.434315e-01 | ko00130 |
| 65 | C5-Branched dibasic acid metabolism | 1 | 8 | 0.3722707 | 6.643600e-01 | ko00660 |
| 66 | Propanoate metabolism | 6 | 89 | 0.3896908 | 6.849111e-01 | ko00640 |
| 67 | Riboflavin metabolism | 3 | 41 | 0.4111955 | 7.119206e-01 | ko00740 |
| 68 | Phenylalanine, tyrosine and tryptophan biosynthesis | 6 | 92 | 0.4203462 | 7.170612e-01 | ko00400 |
| 69 | Valine, leucine and isoleucine biosynthesis | 7 | 111 | 0.4391866 | 7.383427e-01 | ko00290 |
| 70 | Arachidonic acid metabolism | 2 | 28 | 0.4751796 | 7.874405e-01 | ko00590 |
| 71 | Spliceosome | 39 | 683 | 0.4982875 | 8.141035e-01 | ko03040 |
| 72 | Protein processing in endoplasmic reticulum | 30 | 531 | 0.5288118 | 8.303728e-01 | ko04141 |
| 73 | Sphingolipid metabolism | 4 | 67 | 0.5289764 | 8.303728e-01 | ko00600 |
| 74 | Sesquiterpenoid and triterpenoid biosynthesis | 2 | 31 | 0.5297206 | 8.303728e-01 | ko00909 |
| 75 | Carbon fixation in photosynthetic organisms | 7 | 123 | 0.5479582 | 8.475087e-01 | ko00710 |
| 76 | Thiamine metabolism | 1 | 14 | 0.55736 | 8.507074e-01 | ko00730 |
| 77 | beta-Alanine metabolism | 4 | 71 | 0.5753342 | 8.667372e-01 | ko00410 |
| 78 | Protein export | 5 | 93 | 0.6100458 | 8.953121e-01 | ko03060 |
| 79 | Peroxisome | 8 | 149 | 0.6105654 | 8.953121e-01 | ko04146 |
| 80 | Circadian rhythm - plant | 10 | 187 | 0.6174566 | 8.953121e-01 | ko04712 |
| 81 | Oxidative phosphorylation | 14 | 266 | 0.6473192 | 9.270250e-01 | ko00190 |
| 82 | Base excision repair | 7 | 138 | 0.6693059 | 9.468230e-01 | ko03410 |
| 83 | Pantothenate and CoA biosynthesis | 4 | 82 | 0.6882747 | 9.508985e-01 | ko00770 |
| 84 | Fatty acid elongation | 3 | 62 | 0.6885817 | 9.508985e-01 | ko00062 |
| 85 | Pentose phosphate pathway | 5 | 103 | 0.6987112 | 9.535353e-01 | ko00030 |
| 86 | Valine, leucine and isoleucine degradation | 5 | 105 | 0.7146579 | 9.639572e-01 | ko00280 |
| 87 | Arginine and proline metabolism | 6 | 127 | 0.7301451 | 9.715100e-01 | ko00330 |
| 88 | Porphyrin and chlorophyll metabolism | 6 | 128 | 0.7370076 | 9.715100e-01 | ko00860 |
| 89 | Nucleotide excision repair | 8 | 171 | 0.757071 | 9.867442e-01 | ko03420 |
| 90 | Phosphatidylinositol signaling system | 6 | 140 | 0.8097151 | 1.000000e+00 | ko04070 |
| 91 | Photosynthesis - antenna proteins | 1 | 29 | 0.8152629 | 1.000000e+00 | ko00196 |
| 92 | Proteasome | 3 | 77 | 0.8180068 | 1.000000e+00 | ko03050 |
| 93 | DNA replication | 5 | 122 | 0.8261808 | 1.000000e+00 | ko03030 |
| 94 | Glycosylphosphatidylinositol(GPI)-anchor biosynthesis | 4 | 115 | 0.8957725 | 1.000000e+00 | ko00563 |
| 95 | Phagosome | 8 | 204 | 0.8959992 | 1.000000e+00 | ko04145 |
| 96 | Sulfur metabolism | 1 | 42 | 0.9134165 | 1.000000e+00 | ko00920 |
| 97 | Glycine, serine and threonine metabolism | 6 | 168 | 0.9183925 | 1.000000e+00 | ko00260 |
| 98 | Steroid biosynthesis | 2 | 72 | 0.9198615 | 1.000000e+00 | ko00100 |
| 99 | Citrate cycle (TCA cycle) | 2 | 73 | 0.9235514 | 1.000000e+00 | ko00020 |
| 100 | Terpenoid backbone biosynthesis | 3 | 115 | 0.9613877 | 1.000000e+00 | ko00900 |
| 101 | Lysine biosynthesis | 1 | 71 | 0.9840595 | 1.000000e+00 | ko00300 |
| 102 | Inositol phosphate metabolism | 3 | 139 | 0.9867785 | 1.000000e+00 | ko00562 |
| 103 | N-Glycan biosynthesis | 1 | 76 | 0.9880962 | 1.000000e+00 | ko00510 |
| 104 | SNARE interactions in vesicular transport | 1 | 82 | 0.9916157 | 1.000000e+00 | ko04130 |
| 105 | Natural killer cell mediated cytotoxicity | 1 | 87 | 0.99374 | 1.000000e+00 | ko04650 |
| 106 | Basal transcription factors | 2 | 129 | 0.9952902 | 1.000000e+00 | ko03022 |
| 107 | Photosynthesis | 1 | 101 | 0.9972385 | 1.000000e+00 | ko00195 |
| 108 | Aminoacyl-tRNA biosynthesis | 2 | 147 | 0.998155 | 1.000000e+00 | ko00970 |
| 109 | Mismatch repair | 1 | 110 | 0.9983688 | 1.000000e+00 | ko03430 |
| 110 | Homologous recombination | 6 | 277 | 0.9986613 | 1.000000e+00 | ko03440 |
| 111 | Ribosome | 10 | 395 | 0.999116 | 1.000000e+00 | ko03010 |
| 112 | Ubiquitin mediated proteolysis | 8 | 376 | 0.9997746 | 1.000000e+00 | ko04120 |
| 113 | mRNA surveillance pathway | 18 | 674 | 0.9999405 | 1.000000e+00 | ko03015 |
| 114 | RNA transport | 36 | 1179 | 0.999994 | 1.000000e+00 | ko03013 |
| 115 | Ribosome biogenesis in eukaryotes | 11 | 592 | 0.999999 | 1.000000e+00 | ko03008 |
| 116 | RNA degradation | 8 | 629 | 1 | 1.000000e+00 | ko03018 |

| # | Pathway | Differentially expressed genes |
| --- | --- | --- |
| 1 | Biosynthesis of secondary metabolites (no map in kegg database) | Unigene14084\_D2, CL5627.Contig2\_D2, CL8008.Contig1\_D2, Unigene17065\_D2, CL2446.Contig4\_D2, Unigene18939\_D2, CL6535.Contig2\_D2, Unigene13213\_D2, Unigene24809\_D2, Unigene12096\_D2, Unigene17066\_D2, Unigene17858\_D2, Unigene30736\_D2, Unigene23818\_D2, Unigene20801\_D2, Unigene7945\_D2, CL6194.Contig1\_D2, Unigene13640\_D2, Unigene4153\_D2, Unigene24537\_D2, Unigene10212\_D2, CL1565.Contig2\_D2, CL5255.Contig1\_D2, CL7899.Contig1\_D2, Unigene12494\_D2, CL2446.Contig3\_D2, CL684.Contig2\_D2, CL1845.Contig2\_D2, Unigene18591\_D2, Unigene17475\_D2, CL7935.Contig1\_D2, CL3914.Contig2\_D2, CL2260.Contig1\_D2, CL5859.Contig1\_D2, Unigene9218\_D2, CL654.Contig4\_D2, Unigene26252\_D2, CL2446.Contig2\_D2, Unigene12683\_D2, CL2568.Contig2\_D2, CL8212.Contig1\_D2, Unigene26284\_D2, CL746.Contig2\_D2, CL1534.Contig1\_D2, CL4906.Contig1\_D2, CL6128.Contig1\_D2, Unigene21218\_D2, CL7437.Contig2\_D2, CL507.Contig3\_D2, Unigene12684\_D2, Unigene21009\_D2, Unigene6452\_D2, CL654.Contig3\_D2, Unigene21645\_D2, Unigene858\_D2, CL1281.Contig2\_D2, Unigene20289\_D2, CL8.Contig2\_D2, Unigene21219\_D2, Unigene15522\_D2, Unigene18442\_D2, Unigene520\_D2, CL840.Contig2\_D2, CL654.Contig1\_D2, Unigene4734\_D2, Unigene17861\_D2, CL2446.Contig1\_D2, CL888.Contig1\_D2, Unigene11409\_D2, Unigene21020\_D2, Unigene24034\_D2, CL1462.Contig3\_D2, Unigene21008\_D2, Unigene7206\_D2, CL5627.Contig1\_D2, Unigene7034\_D2, Unigene24536\_D2, Unigene30431\_D2, Unigene21838\_D2, Unigene16482\_D2, CL795.Contig1\_D2, Unigene1498\_D2, Unigene22153\_D2, Unigene24936\_D2, Unigene15618\_D2, Unigene17776\_D2, CL746.Contig1\_D2, Unigene19581\_D2, CL3914.Contig1\_D2, CL2240.Contig1\_D2, CL507.Contig2\_D2, CL1772.Contig2\_D2, CL2130.Contig1\_D2, CL6192.Contig2\_D2, CL51.Contig2\_D2, Unigene18910\_D2, Unigene1195\_D2, CL331.Contig3\_D2, CL1595.Contig1\_D2, Unigene22154\_D2, Unigene1584\_D2, Unigene17522\_D2, CL5646.Contig2\_D2, Unigene16800\_D2, Unigene30174\_D2, Unigene14272\_D2, Unigene17419\_D2, Unigene21837\_D2, CL7437.Contig1\_D2, Unigene16827\_D2, CL2204.Contig2\_D2, CL154.Contig1\_D2, Unigene487\_D2, Unigene26418\_D2, Unigene21041\_D2, CL3178.Contig1\_D2, Unigene21137\_D2, Unigene27382\_D2, Unigene18956\_D2, Unigene28947\_D2, CL5532.Contig2\_D2, Unigene20266\_D2, Unigene21040\_D2, Unigene17189\_D2, Unigene26466\_D2, CL6302.Contig2\_D2, Unigene26341\_D2, Unigene23531\_D2, CL4739.Contig3\_D2, Unigene28919\_D2, Unigene19863\_D2, Unigene19434\_D2, CL7073.Contig2\_D2, CL3177.Contig1\_D2, Unigene16038\_D2, Unigene1705\_D2, Unigene26338\_D2, CL5475.Contig2\_D2, Unigene26765\_D2, CL4671.Contig2\_D2, Unigene23375\_D2, Unigene25024\_D2, Unigene23223\_D2, Unigene30308\_D2, CL1791.Contig1\_D2, Unigene23473\_D2, Unigene24567\_D2, CL7004.Contig1\_D2, Unigene25561\_D2, Unigene17154\_D2, Unigene25613\_D2, Unigene25085\_D2, CL1918.Contig3\_D2, CL2515.Contig3\_D2, Unigene17123\_D2, Unigene27383\_D2, CL5327.Contig1\_D2, Unigene22564\_D2, Unigene25614\_D2, Unigene24069\_D2, Unigene1207\_D2, CL2975.Contig2\_D2, Unigene21335\_D2, CL5651.Contig1\_D2, CL2939.Contig1\_D2, CL2231.Contig1\_D2, CL6804.Contig2\_D2, Unigene13449\_D2, Unigene14670\_D2, Unigene15282\_D2, CL1087.Contig1\_D2, Unigene4321\_D2, CL4555.Contig2\_D2, Unigene23318\_D2, Unigene16008\_D2, Unigene20806\_D2, Unigene26849\_D2, CL769.Contig2\_D2, CL8143.Contig2\_D2, CL654.Contig5\_D2, CL5646.Contig1\_D2, Unigene28909\_D2, Unigene362\_D2, CL3848.Contig2\_D2, Unigene13058\_D2, Unigene19511\_D2, Unigene13153\_D2, Unigene19512\_D2, CL1842.Contig1\_D2, CL196.Contig6\_D2, Unigene14113\_D2, CL5861.Contig1\_D2, CL2708.Contig2\_D2, Unigene18963\_D2, Unigene28008\_D2, CL5792.Contig1\_D2, CL5311.Contig1\_D2, CL844.Contig1\_D2, Unigene20642\_D2, CL22.Contig5\_D2, CL1842.Contig2\_D2, CL7199.Contig3\_D2, Unigene17483\_D2, Unigene373\_D2, CL7108.Contig1\_D2, CL1077.Contig2\_D2, CL2651.Contig2\_D2, CL6913.Contig1\_D2, Unigene21945\_D2, CL3101.Contig2\_D2, CL3110.Contig1\_D2, Unigene22441\_D2, Unigene18354\_D2, Unigene19948\_D2, CL3729.Contig1\_D2, Unigene26161\_D2, Unigene233\_D2, Unigene25493\_D2, Unigene1955\_D2, CL3848.Contig3\_D2, Unigene27351\_D2, CL5792.Contig3\_D2, Unigene24448\_D2, Unigene30530\_D2, Unigene28931\_D2, CL2630.Contig1\_D2, Unigene19815\_D2, CL2558.Contig1\_D2 |
| 2 | Metabolic pathways (no map in kegg database) | Unigene4819\_D2, Unigene14084\_D2, CL5627.Contig2\_D2, Unigene12242\_D2, CL163.Contig1\_D2, CL8008.Contig1\_D2, Unigene17065\_D2, Unigene13212\_D2, CL678.Contig1\_D2, Unigene31118\_D2, CL6535.Contig2\_D2, Unigene13213\_D2, Unigene24809\_D2, CL7895.Contig2\_D2, Unigene12096\_D2, Unigene17066\_D2, CL3793.Contig2\_D2, CL7895.Contig1\_D2, Unigene12967\_D2, CL2569.Contig2\_D2, CL3338.Contig2\_D2, Unigene28076\_D2, Unigene17858\_D2, CL7212.Contig2\_D2, CL6808.Contig3\_D2, Unigene4690\_D2, Unigene30736\_D2, Unigene23818\_D2, Unigene20801\_D2, CL179.Contig2\_D2, CL780.Contig1\_D2, CL6194.Contig1\_D2, Unigene30089\_D2, Unigene13640\_D2, Unigene4153\_D2, Unigene24537\_D2, Unigene4567\_D2, Unigene10212\_D2, CL288.Contig1\_D2, CL2521.Contig1\_D2, CL7899.Contig1\_D2, CL124.Contig2\_D2, CL684.Contig2\_D2, CL1845.Contig2\_D2, Unigene14386\_D2, Unigene18591\_D2, CL4088.Contig1\_D2, Unigene17475\_D2, CL7935.Contig1\_D2, Unigene21547\_D2, Unigene1884\_D2, CL3914.Contig2\_D2, CL2260.Contig1\_D2, CL678.Contig2\_D2, CL288.Contig2\_D2, CL654.Contig4\_D2, Unigene26252\_D2, Unigene4166\_D2, Unigene13466\_D2, CL288.Contig3\_D2, Unigene12683\_D2, Unigene22358\_D2, Unigene17972\_D2, CL2568.Contig2\_D2, CL8212.Contig1\_D2, Unigene23303\_D2, CL2320.Contig2\_D2, Unigene31520\_D2, Unigene28077\_D2, Unigene26284\_D2, CL746.Contig2\_D2, CL1534.Contig1\_D2, Unigene13444\_D2, CL6128.Contig1\_D2, Unigene21218\_D2, CL7437.Contig2\_D2, CL507.Contig3\_D2, Unigene12684\_D2, Unigene7514\_D2, Unigene21009\_D2, Unigene6452\_D2, CL654.Contig3\_D2, CL1895.Contig1\_D2, Unigene21645\_D2, CL3338.Contig1\_D2, CL5558.Contig2\_D2, Unigene21643\_D2, Unigene858\_D2, CL1281.Contig2\_D2, Unigene20289\_D2, Unigene10071\_D2, Unigene4719\_D2, CL8.Contig2\_D2, CL630.Contig1\_D2, Unigene21219\_D2, Unigene1782\_D2, Unigene15522\_D2, Unigene18442\_D2, Unigene520\_D2, CL840.Contig2\_D2, Unigene8969\_D2, CL654.Contig1\_D2, CL7066.Contig1\_D2, Unigene13073\_D2, Unigene16279\_D2, Unigene4734\_D2, Unigene17861\_D2, CL476.Contig1\_D2, Unigene30062\_D2, Unigene22386\_D2, Unigene22975\_D2, Unigene5776\_D2, Unigene16635\_D2, Unigene11409\_D2, CL1239.Contig1\_D2, Unigene21020\_D2, Unigene17503\_D2, CL1462.Contig3\_D2, Unigene21008\_D2, Unigene7206\_D2, Unigene19328\_D2, CL5627.Contig1\_D2, CL1211.Contig1\_D2, Unigene23775\_D2, Unigene7034\_D2, CL5039.Contig2\_D2, Unigene17140\_D2, Unigene16692\_D2, CL788.Contig3\_D2, Unigene24536\_D2, Unigene6879\_D2, Unigene16411\_D2, Unigene30431\_D2, Unigene19385\_D2, Unigene25049\_D2, Unigene15066\_D2, Unigene21838\_D2, Unigene16482\_D2, CL818.Contig1\_D2, Unigene28965\_D2, CL306.Contig2\_D2, CL795.Contig1\_D2, Unigene1498\_D2, Unigene22153\_D2, Unigene11325\_D2, Unigene24936\_D2, Unigene15618\_D2, Unigene27473\_D2, Unigene1612\_D2, CL469.Contig1\_D2, Unigene4771\_D2, CL746.Contig1\_D2, Unigene17865\_D2, Unigene22630\_D2, CL3914.Contig1\_D2, CL4864.Contig2\_D2, CL2240.Contig1\_D2, CL507.Contig2\_D2, CL1772.Contig2\_D2, CL1267.Contig1\_D2, CL6192.Contig2\_D2, CL51.Contig2\_D2, Unigene18910\_D2, CL331.Contig3\_D2, CL1595.Contig1\_D2, CL479.Contig1\_D2, Unigene22154\_D2, CL5058.Contig1\_D2, Unigene11193\_D2, CL268.Contig1\_D2, Unigene16511\_D2, CL5734.Contig1\_D2, Unigene17522\_D2, Unigene28152\_D2, CL5646.Contig2\_D2, Unigene16800\_D2, Unigene30174\_D2, Unigene14272\_D2, Unigene21837\_D2, CL207.Contig1\_D2, CL7437.Contig1\_D2, CL2204.Contig2\_D2, Unigene20589\_D2, CL154.Contig1\_D2, Unigene21093\_D2, CL3984.Contig2\_D2, CL8112.Contig1\_D2, Unigene26418\_D2, Unigene21041\_D2, CL163.Contig3\_D2, Unigene16603\_D2, Unigene4685\_D2, CL3178.Contig1\_D2, Unigene16674\_D2, Unigene21137\_D2, Unigene23387\_D2, CL4321.Contig1\_D2, Unigene27382\_D2, CL1988.Contig3\_D2, Unigene20991\_D2, Unigene28947\_D2, CL7387.Contig2\_D2, Unigene4720\_D2, Unigene17010\_D2, CL5532.Contig2\_D2, Unigene20266\_D2, Unigene21040\_D2, Unigene17189\_D2, Unigene26466\_D2, Unigene30197\_D2, CL6302.Contig2\_D2, Unigene26341\_D2, Unigene23531\_D2, Unigene28919\_D2, Unigene14202\_D2, Unigene25022\_D2, Unigene23330\_D2, Unigene19022\_D2, Unigene18096\_D2, CL7748.Contig1\_D2, CL2320.Contig3\_D2, Unigene19434\_D2, CL5892.Contig1\_D2, Unigene27428\_D2, CL7073.Contig2\_D2, CL3177.Contig1\_D2, CL1935.Contig1\_D2, Unigene16038\_D2, Unigene27741\_D2, Unigene1675\_D2, Unigene1705\_D2, Unigene26338\_D2, Unigene19375\_D2, Unigene25711\_D2, CL5475.Contig2\_D2, Unigene13479\_D2, CL6311.Contig1\_D2, Unigene26765\_D2, CL4671.Contig2\_D2, Unigene23072\_D2, Unigene23375\_D2, Unigene30350\_D2, CL5574.Contig1\_D2, Unigene16035\_D2, Unigene23053\_D2, Unigene25024\_D2, Unigene19446\_D2, Unigene23223\_D2, Unigene13618\_D2, Unigene26\_D2, Unigene30308\_D2, Unigene29620\_D2, Unigene29326\_D2, CL3078.Contig2\_D2, Unigene18746\_D2, Unigene23473\_D2, CL6487.Contig2\_D2, Unigene24567\_D2, CL7004.Contig1\_D2, Unigene25561\_D2, Unigene17154\_D2, Unigene19825\_D2, Unigene23515\_D2, Unigene20456\_D2, Unigene25613\_D2, Unigene21161\_D2, CL2515.Contig3\_D2, Unigene13568\_D2, Unigene17123\_D2, Unigene1860\_D2, Unigene25801\_D2, CL5327.Contig1\_D2, Unigene29588\_D2, Unigene22564\_D2, CL3878.Contig1\_D2, Unigene25614\_D2, Unigene24069\_D2, Unigene21335\_D2, Unigene21151\_D2, Unigene21542\_D2, Unigene21782\_D2, CL2939.Contig1\_D2, Unigene23935\_D2, Unigene28633\_D2, CL2945.Contig2\_D2, CL1035.Contig2\_D2, Unigene5047\_D2, CL7748.Contig7\_D2, Unigene31639\_D2, CL562.Contig3\_D2, CL6804.Contig2\_D2, Unigene13449\_D2, Unigene14670\_D2, Unigene15282\_D2, Unigene30116\_D2, CL4250.Contig3\_D2, CL3249.Contig2\_D2, CL1087.Contig1\_D2, Unigene4321\_D2, CL4555.Contig2\_D2, Unigene17739\_D2, Unigene25192\_D2, Unigene23318\_D2, Unigene20806\_D2, Unigene26849\_D2, CL4932.Contig1\_D2, CL769.Contig2\_D2, Unigene30760\_D2, CL1718.Contig2\_D2, CL7889.Contig1\_D2, CL654.Contig5\_D2, Unigene11272\_D2, CL5646.Contig1\_D2, Unigene22778\_D2, Unigene362\_D2, Unigene284\_D2, CL3502.Contig2\_D2, CL3848.Contig2\_D2, Unigene13058\_D2, CL6431.Contig2\_D2, Unigene1197\_D2, Unigene17621\_D2, Unigene4783\_D2, Unigene22779\_D2, Unigene22073\_D2, Unigene28146\_D2, Unigene19511\_D2, Unigene30469\_D2, Unigene15732\_D2, Unigene27337\_D2, Unigene13153\_D2, Unigene19512\_D2, CL8049.Contig1\_D2, CL1842.Contig1\_D2, Unigene26042\_D2, Unigene14113\_D2, CL5861.Contig1\_D2, Unigene25595\_D2, CL2708.Contig2\_D2, CL7336.Contig1\_D2, Unigene18963\_D2, Unigene11046\_D2, CL7633.Contig1\_D2, Unigene18479\_D2, Unigene28008\_D2, CL5792.Contig1\_D2, CL5311.Contig1\_D2, CL3212.Contig4\_D2, CL2303.Contig2\_D2, CL844.Contig1\_D2, Unigene21359\_D2, Unigene20642\_D2, CL4596.Contig2\_D2, CL22.Contig5\_D2, CL1842.Contig2\_D2, CL1451.Contig1\_D2, Unigene20189\_D2, CL7199.Contig3\_D2, Unigene17483\_D2, CL3294.Contig2\_D2, Unigene373\_D2, Unigene29072\_D2, CL7108.Contig1\_D2, CL459.Contig2\_D2, CL1211.Contig2\_D2, Unigene19230\_D2, CL1077.Contig2\_D2, CL5998.Contig3\_D2, CL6913.Contig1\_D2, Unigene21945\_D2, CL3101.Contig2\_D2, Unigene17489\_D2, Unigene28221\_D2, CL3110.Contig1\_D2, Unigene22441\_D2, Unigene21362\_D2, Unigene18354\_D2, Unigene19948\_D2, CL3729.Contig1\_D2, Unigene26161\_D2, Unigene233\_D2, Unigene25493\_D2, Unigene20119\_D2, Unigene1955\_D2, CL2248.Contig1\_D2, Unigene21902\_D2, CL3848.Contig3\_D2, CL6989.Contig2\_D2, CL5792.Contig3\_D2, Unigene24448\_D2, Unigene22085\_D2, Unigene26454\_D2, Unigene25797\_D2, Unigene18285\_D2, Unigene20120\_D2, Unigene1792\_D2, Unigene28931\_D2, Unigene26897\_D2, CL7909.Contig2\_D2, Unigene17657\_D2, Unigene24105\_D2, CL7143.Contig2\_D2, Unigene25931\_D2, CL2630.Contig1\_D2, Unigene29448\_D2, Unigene19815\_D2, Unigene26596\_D2 |
| 3 | Phenylpropanoid biosynthesis | CL5627.Contig2\_D2, CL8008.Contig1\_D2, Unigene12096\_D2, Unigene17858\_D2, Unigene30736\_D2, Unigene20801\_D2, Unigene24537\_D2, Unigene10212\_D2, CL1845.Contig2\_D2, Unigene17475\_D2, CL3914.Contig2\_D2, CL654.Contig4\_D2, Unigene12683\_D2, CL746.Contig2\_D2, Unigene21218\_D2, Unigene12684\_D2, Unigene21009\_D2, CL654.Contig3\_D2, Unigene858\_D2, Unigene21219\_D2, Unigene18442\_D2, Unigene520\_D2, CL654.Contig1\_D2, Unigene4734\_D2, Unigene17861\_D2, CL888.Contig1\_D2, Unigene21020\_D2, CL5822.Contig1\_D2, Unigene21008\_D2, CL5627.Contig1\_D2, Unigene16482\_D2, Unigene29239\_D2, CL746.Contig1\_D2, CL3914.Contig1\_D2, CL2240.Contig1\_D2, CL1867.Contig1\_D2, CL5646.Contig2\_D2, Unigene16800\_D2, Unigene14272\_D2, Unigene28947\_D2, CL2403.Contig2\_D2, Unigene20266\_D2, CL6302.Contig2\_D2, Unigene23531\_D2, Unigene26765\_D2, Unigene25613\_D2, Unigene27383\_D2, CL2975.Contig2\_D2, Unigene4321\_D2, Unigene30341\_D2, Unigene16008\_D2, Unigene26849\_D2, CL8143.Contig2\_D2, CL654.Contig5\_D2, CL5646.Contig1\_D2, Unigene28909\_D2, Unigene362\_D2, CL4187.Contig1\_D2, CL3848.Contig2\_D2, CL5737.Contig2\_D2, CL1842.Contig1\_D2, CL1842.Contig2\_D2, CL3110.Contig1\_D2, Unigene25493\_D2, CL3848.Contig3\_D2, CL5792.Contig3\_D2 |
| 4 | Phenylalanine metabolism | CL5627.Contig2\_D2, Unigene17858\_D2, Unigene30736\_D2, Unigene20801\_D2, Unigene24537\_D2, Unigene17475\_D2, Unigene12683\_D2, CL746.Contig2\_D2, Unigene12684\_D2, Unigene21009\_D2, Unigene858\_D2, Unigene18442\_D2, Unigene520\_D2, CL840.Contig2\_D2, Unigene4734\_D2, Unigene17861\_D2, Unigene21020\_D2, Unigene21008\_D2, CL5627.Contig1\_D2, Unigene16482\_D2, CL746.Contig1\_D2, CL5646.Contig2\_D2, Unigene14272\_D2, Unigene28947\_D2, Unigene20266\_D2, Unigene25613\_D2, Unigene4321\_D2, CL5646.Contig1\_D2, CL3848.Contig2\_D2, CL1842.Contig1\_D2, CL1842.Contig2\_D2, CL7199.Contig3\_D2, Unigene18354\_D2, CL3848.Contig3\_D2, CL5792.Contig3\_D2 |
| 5 | Plant-pathogen interaction | CL5727.Contig2\_D2, Unigene12404\_D2, CL9.Contig2\_D2, Unigene7948\_D2, CL3552.Contig1\_D2, CL380.Contig1\_D2, Unigene12405\_D2, Unigene19809\_D2, Unigene4231\_D2, CL678.Contig1\_D2, Unigene31951\_D2, CL7895.Contig2\_D2, CL2734.Contig5\_D2, CL7895.Contig1\_D2, CL7866.Contig1\_D2, Unigene17355\_D2, Unigene11533\_D2, CL4809.Contig1\_D2, CL2521.Contig1\_D2, Unigene26445\_D2, CL124.Contig2\_D2, CL2734.Contig2\_D2, Unigene4392\_D2, Unigene4380\_D2, Unigene58\_D2, Unigene18967\_D2, Unigene8896\_D2, CL678.Contig2\_D2, CL2734.Contig1\_D2, Unigene17343\_D2, Unigene18029\_D2, CL6389.Contig1\_D2, CL3454.Contig1\_D2, CL6015.Contig1\_D2, Unigene15841\_D2, Unigene30694\_D2, CL552.Contig2\_D2, CL26.Contig1\_D2, Unigene24564\_D2, Unigene18618\_D2, CL826.Contig1\_D2, CL2734.Contig3\_D2, CL630.Contig1\_D2, CL7366.Contig1\_D2, Unigene14333\_D2, CL7780.Contig1\_D2, Unigene8901\_D2, CL1409.Contig3\_D2, Unigene13073\_D2, CL4368.Contig2\_D2, CL7904.Contig1\_D2, CL3381.Contig1\_D2, Unigene678\_D2, CL4771.Contig2\_D2, CL2923.Contig1\_D2, Unigene1400\_D2, CL788.Contig3\_D2, Unigene17822\_D2, Unigene7\_D2, Unigene14247\_D2, Unigene15066\_D2, CL7619.Contig1\_D2, CL9.Contig1\_D2, Unigene4256\_D2, Unigene18675\_D2, CL2734.Contig4\_D2, Unigene391\_D2, Unigene1405\_D2, CL4849.Contig2\_D2, Unigene30249\_D2, CL1160.Contig2\_D2, Unigene21792\_D2, CL705.Contig2\_D2, CL1267.Contig1\_D2, Unigene1122\_D2, Unigene27055\_D2, CL268.Contig1\_D2, CL4849.Contig1\_D2, Unigene18174\_D2, CL1332.Contig3\_D2, Unigene15524\_D2, Unigene21150\_D2, Unigene18513\_D2, Unigene30326\_D2, CL5386.Contig1\_D2, Unigene20674\_D2, Unigene25083\_D2, Unigene4267\_D2, Unigene23011\_D2, CL1106.Contig1\_D2, CL6400.Contig1\_D2, Unigene17152\_D2, Unigene20562\_D2, CL512.Contig1\_D2, Unigene21876\_D2, CL7748.Contig1\_D2, CL5950.Contig1\_D2, Unigene20326\_D2, Unigene29406\_D2, CL5748.Contig1\_D2, Unigene23004\_D2, Unigene14989\_D2, Unigene20558\_D2, Unigene21550\_D2, Unigene26162\_D2, Unigene18376\_D2, CL7939.Contig1\_D2, Unigene22581\_D2, Unigene26770\_D2, Unigene25467\_D2, Unigene20790\_D2, CL705.Contig1\_D2, CL7748.Contig7\_D2, CL257.Contig6\_D2, CL257.Contig3\_D2, CL2251.Contig3\_D2, CL7889.Contig1\_D2, CL3163.Contig3\_D2, Unigene22164\_D2, Unigene30469\_D2, CL2586.Contig2\_D2, CL8049.Contig1\_D2, CL552.Contig1\_D2, CL7336.Contig1\_D2, Unigene22473\_D2, Unigene28132\_D2, Unigene11094\_D2, Unigene19840\_D2, CL1634.Contig1\_D2, Unigene22162\_D2, Unigene28043\_D2 |
| 6 | Flavone and flavonol biosynthesis | CL8008.Contig1\_D2, Unigene10212\_D2, CL3914.Contig2\_D2, CL2260.Contig1\_D2, CL2568.Contig2\_D2, CL1534.Contig1\_D2, Unigene21218\_D2, Unigene20289\_D2, Unigene21219\_D2, CL1462.Contig3\_D2, Unigene7206\_D2, Unigene29239\_D2, CL3914.Contig1\_D2, CL2240.Contig1\_D2, Unigene20425\_D2, Unigene16800\_D2, Unigene487\_D2, Unigene22912\_D2, Unigene26341\_D2, Unigene16638\_D2, Unigene22619\_D2, Unigene22618\_D2, Unigene6835\_D2, CL3892.Contig1\_D2, CL7857.Contig1\_D2, CL226.Contig1\_D2, Unigene13862\_D2, Unigene13664\_D2, CL5861.Contig1\_D2, Unigene23310\_D2 |
| 7 | Flavonoid biosynthesis | CL8008.Contig1\_D2, Unigene26252\_D2, CL2568.Contig2\_D2, CL1534.Contig1\_D2, Unigene21645\_D2, Unigene20289\_D2, CL1462.Contig3\_D2, Unigene7206\_D2, Unigene19581\_D2, CL1772.Contig2\_D2, CL2130.Contig1\_D2, CL51.Contig2\_D2, Unigene18910\_D2, CL1595.Contig1\_D2, Unigene17522\_D2, Unigene487\_D2, CL5532.Contig2\_D2, Unigene26341\_D2, Unigene26338\_D2, CL7004.Contig1\_D2, Unigene25561\_D2, Unigene27383\_D2, Unigene1207\_D2, CL2975.Contig2\_D2, CL5651.Contig1\_D2, CL6804.Contig2\_D2, CL4555.Contig2\_D2, Unigene16008\_D2, CL769.Contig2\_D2, CL8143.Contig2\_D2, Unigene28909\_D2, CL3848.Contig2\_D2, CL5861.Contig1\_D2, CL3101.Contig2\_D2, Unigene22441\_D2, CL3848.Contig3\_D2 |
| 8 | alpha-Linolenic acid metabolism | Unigene4819\_D2, Unigene12967\_D2, CL3338.Contig2\_D2, CL179.Contig2\_D2, Unigene30089\_D2, CL288.Contig1\_D2, CL6608.Contig1\_D2, CL4088.Contig1\_D2, Unigene8880\_D2, CL288.Contig2\_D2, CL288.Contig3\_D2, CL2320.Contig2\_D2, CL3338.Contig1\_D2, Unigene18442\_D2, Unigene22950\_D2, CL7066.Contig1\_D2, Unigene19328\_D2, Unigene22951\_D2, CL479.Contig1\_D2, Unigene28152\_D2, Unigene7707\_D2, CL2320.Contig3\_D2, CL5475.Contig2\_D2, Unigene1860\_D2, Unigene31595\_D2, CL5792.Contig1\_D2, CL1131.Contig1\_D2, CL7143.Contig2\_D2 |
| 9 | Stilbenoid, diarylheptanoid and gingerol biosynthesis | CL8008.Contig1\_D2, CL6535.Contig2\_D2, Unigene13640\_D2, CL7899.Contig1\_D2, Unigene18591\_D2, CL2568.Contig2\_D2, CL1534.Contig1\_D2, CL1281.Contig2\_D2, Unigene20289\_D2, CL1462.Contig3\_D2, Unigene7206\_D2, Unigene21838\_D2, Unigene1498\_D2, Unigene21837\_D2, CL2204.Contig2\_D2, Unigene27382\_D2, Unigene26341\_D2, Unigene27383\_D2, CL2975.Contig2\_D2, Unigene16008\_D2, CL8143.Contig2\_D2, Unigene28909\_D2, CL3848.Contig2\_D2, CL5861.Contig1\_D2, CL3848.Contig3\_D2 |
| 10 | Alanine, aspartate and glutamate metabolism | Unigene10071\_D2, CL1211.Contig1\_D2, CL306.Contig2\_D2, CL6192.Contig2\_D2, Unigene21137\_D2, Unigene19434\_D2, CL4671.Contig2\_D2, Unigene16035\_D2, Unigene15282\_D2, Unigene20642\_D2, Unigene17483\_D2, CL1211.Contig2\_D2, Unigene18354\_D2, Unigene20119\_D2, Unigene24448\_D2, Unigene22085\_D2, Unigene20120\_D2, CL7909.Contig2\_D2 |
| 11 | Plant hormone signal transduction | CL5727.Contig2\_D2, Unigene16189\_D2, Unigene19842\_D2, Unigene17355\_D2, Unigene26445\_D2, CL6297.Contig2\_D2, Unigene4380\_D2, Unigene18967\_D2, Unigene31203\_D2, Unigene17343\_D2, Unigene15326\_D2, CL6389.Contig1\_D2, Unigene16261\_D2, Unigene15372\_D2, Unigene19967\_D2, CL7780.Contig1\_D2, CL2293.Contig2\_D2, CL4974.Contig1\_D2, Unigene8901\_D2, CL3676.Contig1\_D2, Unigene15646\_D2, CL6167.Contig2\_D2, Unigene4256\_D2, Unigene17056\_D2, Unigene24620\_D2, Unigene391\_D2, Unigene1405\_D2, CL3888.Contig2\_D2, Unigene12336\_D2, Unigene19979\_D2, CL3319.Contig2\_D2, Unigene17776\_D2, Unigene23364\_D2, Unigene23968\_D2, Unigene13807\_D2, CL102.Contig7\_D2, Unigene26530\_D2, Unigene16678\_D2, Unigene4733\_D2, Unigene18174\_D2, CL1332.Contig3\_D2, CL242.Contig3\_D2, Unigene24622\_D2, Unigene29794\_D2, Unigene26712\_D2, Unigene24623\_D2, CL5386.Contig1\_D2, CL7150.Contig2\_D2, Unigene23011\_D2, Unigene23686\_D2, Unigene14358\_D2, Unigene18326\_D2, Unigene26776\_D2, Unigene22445\_D2, Unigene17152\_D2, CL3739.Contig2\_D2, Unigene20562\_D2, CL512.Contig1\_D2, Unigene16602\_D2, Unigene20326\_D2, Unigene29406\_D2, Unigene23004\_D2, Unigene18522\_D2, Unigene19446\_D2, Unigene21550\_D2, Unigene30189\_D2, CL6767.Contig1\_D2, Unigene1965\_D2, Unigene21587\_D2, Unigene218\_D2, Unigene21151\_D2, Unigene20790\_D2, Unigene28581\_D2, CL6297.Contig3\_D2, Unigene16685\_D2, CL6442.Contig2\_D2, CL4305.Contig2\_D2, CL3163.Contig3\_D2, CL4885.Contig1\_D2, Unigene19054\_D2, CL1498.Contig1\_D2, Unigene13686\_D2, Unigene18976\_D2, Unigene10342\_D2, Unigene18327\_D2, CL1634.Contig1\_D2, Unigene21271\_D2, Unigene19803\_D2, Unigene27787\_D2, Unigene27785\_D2, CL1345.Contig2\_D2, CL6828.Contig2\_D2, Unigene18000\_D2 |
| 12 | Endocytosis | Unigene13212\_D2, CL7212.Contig2\_D2, Unigene4690\_D2, CL1331.Contig1\_D2, Unigene1083\_D2, Unigene14386\_D2, Unigene21547\_D2, Unigene1884\_D2, Unigene17972\_D2, Unigene1727\_D2, Unigene31520\_D2, Unigene13444\_D2, CL5558.Contig2\_D2, CL1331.Contig3\_D2, Unigene4719\_D2, Unigene22386\_D2, Unigene16635\_D2, Unigene17503\_D2, CL283.Contig2\_D2, CL7403.Contig3\_D2, Unigene6879\_D2, Unigene19385\_D2, CL818.Contig1\_D2, CL283.Contig1\_D2, Unigene27473\_D2, Unigene4771\_D2, Unigene22630\_D2, CL7403.Contig1\_D2, Unigene1401\_D2, CL4864.Contig2\_D2, CL5630.Contig1\_D2, Unigene11193\_D2, Unigene16511\_D2, Unigene17485\_D2, CL7403.Contig2\_D2, Unigene4685\_D2, Unigene23387\_D2, CL4321.Contig1\_D2, Unigene596\_D2, Unigene20991\_D2, Unigene4720\_D2, Unigene23330\_D2, CL3177.Contig1\_D2, Unigene1675\_D2, Unigene25711\_D2, Unigene13479\_D2, Unigene23053\_D2, Unigene19446\_D2, Unigene26\_D2, Unigene29326\_D2, CL3078.Contig2\_D2, Unigene19825\_D2, Unigene23515\_D2, Unigene21161\_D2, Unigene13568\_D2, Unigene29588\_D2, Unigene23935\_D2, CL2945.Contig2\_D2, Unigene5047\_D2, Unigene17739\_D2, Unigene30760\_D2, Unigene17621\_D2, Unigene22073\_D2, Unigene25595\_D2, Unigene11046\_D2, Unigene18479\_D2, Unigene21359\_D2, CL4596.Contig2\_D2, Unigene1183\_D2, Unigene29072\_D2, Unigene18103\_D2, Unigene19230\_D2, Unigene26454\_D2, Unigene1792\_D2 |
| 13 | Ether lipid metabolism | Unigene13212\_D2, CL7212.Contig2\_D2, Unigene4690\_D2, Unigene14386\_D2, Unigene21547\_D2, Unigene1884\_D2, Unigene17972\_D2, Unigene31520\_D2, Unigene13444\_D2, CL5558.Contig2\_D2, Unigene4719\_D2, Unigene22386\_D2, Unigene16635\_D2, Unigene17503\_D2, Unigene6879\_D2, Unigene19385\_D2, CL818.Contig1\_D2, Unigene27473\_D2, Unigene4771\_D2, Unigene22630\_D2, CL4864.Contig2\_D2, Unigene11193\_D2, Unigene16511\_D2, Unigene4685\_D2, Unigene23387\_D2, CL4321.Contig1\_D2, Unigene20991\_D2, Unigene4720\_D2, Unigene23330\_D2, CL3177.Contig1\_D2, Unigene27741\_D2, Unigene1675\_D2, Unigene25711\_D2, Unigene13479\_D2, Unigene23053\_D2, Unigene19446\_D2, Unigene26\_D2, Unigene29326\_D2, CL3078.Contig2\_D2, Unigene19825\_D2, Unigene23515\_D2, Unigene13568\_D2, Unigene29588\_D2, Unigene23935\_D2, CL2945.Contig2\_D2, Unigene5047\_D2, Unigene17739\_D2, Unigene30760\_D2, Unigene17621\_D2, Unigene22073\_D2, Unigene25595\_D2, Unigene11046\_D2, Unigene18479\_D2, Unigene21359\_D2, CL4596.Contig2\_D2, Unigene29072\_D2, Unigene19230\_D2, Unigene26454\_D2, Unigene1792\_D2 |
| 14 | Glycerophospholipid metabolism | Unigene4819\_D2, Unigene12242\_D2, Unigene13212\_D2, Unigene12967\_D2, CL3338.Contig2\_D2, CL7212.Contig2\_D2, Unigene4690\_D2, CL179.Contig2\_D2, Unigene14386\_D2, Unigene21547\_D2, Unigene1884\_D2, Unigene17972\_D2, Unigene31520\_D2, Unigene13444\_D2, CL3338.Contig1\_D2, CL5558.Contig2\_D2, Unigene4719\_D2, CL7066.Contig1\_D2, Unigene22386\_D2, Unigene16635\_D2, Unigene17503\_D2, Unigene6879\_D2, Unigene19385\_D2, CL818.Contig1\_D2, Unigene27473\_D2, Unigene4771\_D2, Unigene22630\_D2, CL4864.Contig2\_D2, CL479.Contig1\_D2, Unigene11193\_D2, Unigene16511\_D2, Unigene4685\_D2, Unigene23387\_D2, CL4321.Contig1\_D2, Unigene20991\_D2, Unigene4720\_D2, Unigene23330\_D2, CL3177.Contig1\_D2, Unigene27741\_D2, Unigene1675\_D2, Unigene25711\_D2, Unigene13479\_D2, Unigene23053\_D2, Unigene19446\_D2, Unigene26\_D2, Unigene29326\_D2, CL3078.Contig2\_D2, Unigene19825\_D2, Unigene23515\_D2, Unigene13568\_D2, Unigene25801\_D2, Unigene29588\_D2, Unigene23935\_D2, CL2945.Contig2\_D2, Unigene5047\_D2, Unigene17739\_D2, Unigene30760\_D2, Unigene17621\_D2, Unigene22073\_D2, Unigene25595\_D2, Unigene11046\_D2, Unigene18479\_D2, CL5849.Contig1\_D2, Unigene21359\_D2, CL4596.Contig2\_D2, Unigene27591\_D2, Unigene29072\_D2, Unigene886\_D2, Unigene22498\_D2, Unigene19230\_D2, Unigene26454\_D2, Unigene1792\_D2, Unigene26596\_D2 |
| 15 | Zeatin biosynthesis | CL1565.Contig2\_D2, CL684.Contig2\_D2, Unigene6452\_D2, Unigene9317\_D2, Unigene24034\_D2, Unigene30431\_D2, Unigene29239\_D2, Unigene20425\_D2, Unigene22912\_D2, CL4739.Contig3\_D2, Unigene16638\_D2, Unigene22619\_D2, CL1791.Contig1\_D2, CL1918.Contig3\_D2, CL7426.Contig2\_D2, Unigene22618\_D2, Unigene6835\_D2, CL3892.Contig1\_D2, CL7857.Contig1\_D2, CL226.Contig1\_D2, Unigene13862\_D2, Unigene13664\_D2, Unigene23310\_D2 |
| 16 | Carotenoid biosynthesis | Unigene4575\_D2, Unigene23818\_D2, CL6194.Contig1\_D2, Unigene9133\_D2, Unigene11409\_D2, Unigene7034\_D2, Unigene22153\_D2, Unigene22154\_D2, Unigene16038\_D2, Unigene23375\_D2, Unigene25024\_D2, Unigene30308\_D2, Unigene24567\_D2, Unigene17154\_D2, Unigene14670\_D2, Unigene13153\_D2, Unigene18963\_D2, Unigene28008\_D2, CL1077.Contig2\_D2, Unigene26161\_D2, Unigene1730\_D2 |
| 17 | Cyanoamino acid metabolism | Unigene12096\_D2, Unigene13640\_D2, CL1565.Contig2\_D2, CL654.Contig4\_D2, CL654.Contig3\_D2, CL654.Contig1\_D2, CL888.Contig1\_D2, Unigene24034\_D2, Unigene30431\_D2, Unigene1195\_D2, CL4739.Contig3\_D2, Unigene25022\_D2, Unigene26765\_D2, Unigene30350\_D2, CL1791.Contig1\_D2, CL1918.Contig3\_D2, Unigene21542\_D2, CL2231.Contig1\_D2, Unigene26849\_D2, CL654.Contig5\_D2, Unigene362\_D2 |
| 18 | Limonene and pinene degradation | CL8008.Contig1\_D2, CL6535.Contig2\_D2, Unigene13640\_D2, CL7899.Contig1\_D2, Unigene18591\_D2, CL2568.Contig2\_D2, CL1534.Contig1\_D2, CL1281.Contig2\_D2, Unigene20289\_D2, CL1462.Contig3\_D2, Unigene7206\_D2, Unigene21838\_D2, Unigene1498\_D2, Unigene21837\_D2, CL2204.Contig2\_D2, Unigene27382\_D2, Unigene26341\_D2, CL5861.Contig1\_D2, Unigene30530\_D2 |
| 19 | Fatty acid biosynthesis | CL5946.Contig1\_D2, Unigene17140\_D2, Unigene20589\_D2, Unigene23072\_D2, Unigene25192\_D2, CL3212.Contig4\_D2, CL3294.Contig2\_D2, CL6913.Contig1\_D2, Unigene21902\_D2, CL4581.Contig1\_D2, Unigene17657\_D2 |
| 20 | Other glycan degradation | Unigene23819\_D2, Unigene23818\_D2, Unigene7034\_D2, Unigene28965\_D2, Unigene21041\_D2, CL1988.Contig3\_D2, Unigene21040\_D2, Unigene16038\_D2, Unigene23375\_D2, Unigene25024\_D2, Unigene30308\_D2, CL6487.Contig2\_D2, Unigene17154\_D2, Unigene13153\_D2, Unigene27520\_D2, CL1077.Contig2\_D2, Unigene26161\_D2, CL4234.Contig1\_D2 |
| 21 | ABC transporters | Unigene19227\_D2, CL5655.Contig2\_D2, CL1586.Contig3\_D2, Unigene21501\_D2, CL7780.Contig1\_D2, CL5029.Contig1\_D2, Unigene4641\_D2, Unigene21502\_D2, Unigene30779\_D2, Unigene1903\_D2, Unigene23086\_D2, Unigene6\_D2, Unigene17432\_D2, CL1195.Contig2\_D2, Unigene25014\_D2, Unigene24919\_D2, Unigene390\_D2, Unigene26891\_D2, Unigene28364\_D2, CL1017.Contig2\_D2, CL5485.Contig2\_D2, CL6236.Contig1\_D2, Unigene25412\_D2, CL2508.Contig4\_D2, Unigene21206\_D2, CL820.Contig1\_D2, CL190.Contig1\_D2 |
| 22 | Taurine and hypotaurine metabolism | Unigene21137\_D2, Unigene25022\_D2, Unigene19434\_D2, CL4671.Contig2\_D2, Unigene30350\_D2, Unigene25797\_D2 |
| 23 | Starch and sucrose metabolism | Unigene31118\_D2, Unigene12096\_D2, CL7212.Contig2\_D2, CL654.Contig4\_D2, Unigene13466\_D2, Unigene23303\_D2, CL654.Contig3\_D2, CL5558.Contig2\_D2, CL654.Contig1\_D2, Unigene14451\_D2, Unigene16279\_D2, Unigene30062\_D2, Unigene16635\_D2, CL5039.Contig2\_D2, Unigene16692\_D2, Unigene6879\_D2, CL795.Contig1\_D2, Unigene11325\_D2, CL469.Contig1\_D2, CL3105.Contig1\_D2, Unigene16680\_D2, Unigene16679\_D2, CL5734.Contig1\_D2, Unigene21093\_D2, Unigene4685\_D2, Unigene16674\_D2, Unigene21322\_D2, Unigene21323\_D2, Unigene30197\_D2, Unigene14202\_D2, Unigene26765\_D2, CL5574.Contig1\_D2, Unigene29620\_D2, CL3878.Contig1\_D2, Unigene21782\_D2, Unigene5047\_D2, Unigene26849\_D2, CL654.Contig5\_D2, Unigene362\_D2, Unigene15732\_D2, Unigene18479\_D2, CL1451.Contig1\_D2, Unigene20189\_D2, Unigene4673\_D2, CL459.Contig2\_D2, CL5998.Contig3\_D2, Unigene26454\_D2, Unigene26897\_D2 |
| 24 | Benzoxazinoid biosynthesis | CL1565.Contig2\_D2, Unigene24034\_D2, CL1772.Contig2\_D2, CL5532.Contig2\_D2, CL1791.Contig1\_D2, CL7004.Contig1\_D2, CL2231.Contig1\_D2, CL4555.Contig2\_D2 |
| 25 | Pentose and glucuronate interconversions | CL3793.Contig2\_D2, CL7212.Contig2\_D2, CL2304.Contig1\_D2, Unigene23303\_D2, Unigene21645\_D2, CL5558.Contig2\_D2, Unigene16635\_D2, Unigene16692\_D2, Unigene6879\_D2, CL795.Contig1\_D2, Unigene11325\_D2, CL51.Contig2\_D2, Unigene21093\_D2, Unigene4685\_D2, Unigene16674\_D2, Unigene14202\_D2, CL5574.Contig1\_D2, Unigene21968\_D2, Unigene29620\_D2, Unigene5047\_D2, CL6804.Contig2\_D2, CL769.Contig2\_D2, Unigene15732\_D2, Unigene18479\_D2, Unigene20189\_D2, CL459.Contig2\_D2, CL5998.Contig3\_D2, Unigene26454\_D2 |
| 26 | Glyoxylate and dicarboxylate metabolism | Unigene26284\_D2, CL507.Contig3\_D2, CL306.Contig2\_D2, CL507.Contig2\_D2, Unigene1705\_D2, Unigene27900\_D2, Unigene20806\_D2, Unigene22778\_D2, Unigene22779\_D2, CL7633.Contig1\_D2, Unigene20119\_D2, Unigene20120\_D2, Unigene28931\_D2 |
| 27 | Galactose metabolism | Unigene12096\_D2, Unigene21645\_D2, Unigene30062\_D2, CL5039.Contig2\_D2, Unigene28965\_D2, Unigene24936\_D2, CL51.Contig2\_D2, CL5734.Contig1\_D2, CL1988.Contig3\_D2, Unigene23473\_D2, CL6487.Contig2\_D2, Unigene22\_D2, Unigene901\_D2, CL6804.Contig2\_D2, CL769.Contig2\_D2 |
| 28 | RNA polymerase | CL163.Contig1\_D2, CL678.Contig1\_D2, CL7895.Contig2\_D2, CL7895.Contig1\_D2, CL2569.Contig2\_D2, CL2521.Contig1\_D2, CL124.Contig2\_D2, CL678.Contig2\_D2, Unigene4166\_D2, Unigene7514\_D2, CL1895.Contig1\_D2, CL630.Contig1\_D2, Unigene1782\_D2, Unigene13073\_D2, CL476.Contig1\_D2, CL788.Contig3\_D2, Unigene25049\_D2, Unigene15066\_D2, CL1267.Contig1\_D2, CL268.Contig1\_D2, CL207.Contig1\_D2, CL163.Contig3\_D2, Unigene17010\_D2, CL7748.Contig1\_D2, CL5892.Contig1\_D2, Unigene19375\_D2, Unigene19446\_D2, Unigene21151\_D2, Unigene28633\_D2, CL7748.Contig7\_D2, CL7889.Contig1\_D2, Unigene284\_D2, CL3502.Contig2\_D2, Unigene1197\_D2, Unigene30469\_D2, Unigene27337\_D2, CL8049.Contig1\_D2, CL7336.Contig1\_D2, Unigene29448\_D2 |
| 29 | Diterpenoid biosynthesis | CL8008.Contig1\_D2, CL6194.Contig1\_D2, Unigene13640\_D2, CL1462.Contig3\_D2, Unigene23223\_D2, Unigene14670\_D2, Unigene13058\_D2, Unigene19948\_D2 |
| 30 | Glycosphingolipid biosynthesis - globo series | Unigene21041\_D2, Unigene21040\_D2, Unigene901\_D2 |
| 31 | Indole alkaloid biosynthesis | Unigene26418\_D2, Unigene18956\_D2, CL2651.Contig2\_D2, CL2558.Contig1\_D2 |
| 32 | Tyrosine metabolism | Unigene14084\_D2, CL8212.Contig1\_D2, CL840.Contig2\_D2, CL331.Contig3\_D2, Unigene26466\_D2, CL3177.Contig1\_D2, CL2939.Contig1\_D2, CL1087.Contig1\_D2, CL2708.Contig2\_D2, CL7199.Contig3\_D2, Unigene18354\_D2, CL7909.Contig2\_D2 |
| 33 | Cutin, suberine and wax biosynthesis | CL6189.Contig2\_D2, CL6522.Contig1\_D2, Unigene17439\_D2, Unigene4188\_D2, Unigene9277\_D2, Unigene21838\_D2, Unigene1498\_D2, Unigene21837\_D2, Unigene25085\_D2, Unigene27383\_D2 |
| 34 | Biosynthesis of unsaturated fatty acids | CL5946.Contig1\_D2, Unigene17140\_D2, Unigene28919\_D2, CL5475.Contig2\_D2, Unigene25192\_D2, CL3294.Contig2\_D2, CL4581.Contig1\_D2, CL7143.Contig2\_D2 |
| 35 | Ascorbate and aldarate metabolism | Unigene28076\_D2, CL780.Contig1\_D2, Unigene4567\_D2, Unigene22358\_D2, Unigene28077\_D2, CL6128.Contig1\_D2, Unigene21643\_D2, Unigene22975\_D2, CL795.Contig1\_D2, CL8112.Contig1\_D2, Unigene27428\_D2, Unigene28146\_D2 |
| 36 | Pyrimidine metabolism | CL163.Contig1\_D2, CL678.Contig1\_D2, CL7895.Contig2\_D2, CL7895.Contig1\_D2, CL2569.Contig2\_D2, CL2521.Contig1\_D2, CL124.Contig2\_D2, CL678.Contig2\_D2, Unigene4166\_D2, Unigene7514\_D2, CL1895.Contig1\_D2, Unigene10071\_D2, CL630.Contig1\_D2, Unigene1782\_D2, Unigene13073\_D2, CL476.Contig1\_D2, CL1211.Contig1\_D2, CL788.Contig3\_D2, Unigene25049\_D2, Unigene15066\_D2, CL1267.Contig1\_D2, CL5058.Contig1\_D2, CL268.Contig1\_D2, CL207.Contig1\_D2, CL163.Contig3\_D2, Unigene16603\_D2, Unigene17010\_D2, CL7748.Contig1\_D2, CL5892.Contig1\_D2, Unigene19375\_D2, Unigene16035\_D2, Unigene19446\_D2, Unigene21151\_D2, Unigene28633\_D2, CL7748.Contig7\_D2, CL7889.Contig1\_D2, Unigene284\_D2, CL3502.Contig2\_D2, CL6431.Contig2\_D2, Unigene1197\_D2, Unigene30469\_D2, Unigene27337\_D2, CL8049.Contig1\_D2, CL7336.Contig1\_D2, CL1211.Contig2\_D2, Unigene21362\_D2, Unigene22085\_D2, Unigene18285\_D2, Unigene29448\_D2 |
| 37 | Nitrogen metabolism | CL4602.Contig2\_D2, CL4602.Contig1\_D2, CL306.Contig2\_D2, CL6192.Contig2\_D2, Unigene27900\_D2, Unigene15282\_D2, Unigene20642\_D2, Unigene20119\_D2, Unigene20120\_D2 |
| 38 | Glycolysis / Gluconeogenesis | Unigene14084\_D2, Unigene21645\_D2, Unigene24936\_D2, Unigene15618\_D2, CL51.Contig2\_D2, CL331.Contig3\_D2, Unigene26466\_D2, CL3177.Contig1\_D2, CL5327.Contig1\_D2, CL6804.Contig2\_D2, CL1087.Contig1\_D2, CL769.Contig2\_D2, CL1718.Contig2\_D2, Unigene19511\_D2, Unigene19512\_D2, CL2708.Contig2\_D2, CL7108.Contig1\_D2, Unigene1955\_D2, Unigene27351\_D2 |
| 39 | Tropane, piperidine and pyridine alkaloid biosynthesis | CL840.Contig2\_D2, CL154.Contig1\_D2, Unigene23318\_D2, CL7199.Contig3\_D2, Unigene18354\_D2, CL3729.Contig1\_D2 |
| 40 | Glycosphingolipid biosynthesis - ganglio series | Unigene28965\_D2, Unigene21041\_D2, CL1988.Contig3\_D2, Unigene21040\_D2, CL6487.Contig2\_D2 |
| 41 | Isoquinoline alkaloid biosynthesis | CL8212.Contig1\_D2, CL840.Contig2\_D2, CL2939.Contig1\_D2, CL7199.Contig3\_D2, Unigene18354\_D2 |
| 42 | Glutathione metabolism | Unigene8969\_D2, CL1239.Contig1\_D2, Unigene23775\_D2, CL123.Contig2\_D2, CL1544.Contig1\_D2, CL7387.Contig2\_D2, Unigene25022\_D2, Unigene27314\_D2, Unigene30350\_D2, CL1616.Contig6\_D2, CL1616.Contig5\_D2, Unigene28724\_D2 |
| 43 | Betalain biosynthesis | Unigene30020\_D2 |
| 44 | Glucosinolate biosynthesis (no map in kegg database) | Unigene17065\_D2, Unigene24809\_D2, Unigene17066\_D2 |
| 45 | Tryptophan metabolism | Unigene26284\_D2, CL507.Contig3\_D2, Unigene14534\_D2, CL507.Contig2\_D2, Unigene17456\_D2, CL7235.Contig1\_D2 |
| 46 | Isoflavonoid biosynthesis | Unigene13640\_D2, Unigene17776\_D2, CL1825.Contig4\_D2, CL1825.Contig5\_D2, Unigene24618\_D2 |
| 47 | Butanoate metabolism | Unigene21137\_D2, Unigene19434\_D2, CL4671.Contig2\_D2, Unigene1955\_D2, CL7909.Contig2\_D2 |
| 48 | Amino sugar and nucleotide sugar metabolism | Unigene31118\_D2, CL2446.Contig4\_D2, Unigene7945\_D2, CL5255.Contig1\_D2, Unigene12494\_D2, CL2446.Contig3\_D2, CL5859.Contig1\_D2, Unigene9218\_D2, CL2446.Contig2\_D2, CL4906.Contig1\_D2, CL2446.Contig1\_D2, CL795.Contig1\_D2, Unigene21041\_D2, Unigene21040\_D2, Unigene26765\_D2, Unigene23473\_D2, Unigene362\_D2, Unigene24448\_D2 |
| 49 | Linoleic acid metabolism | Unigene30089\_D2, CL288.Contig1\_D2, CL288.Contig2\_D2, CL288.Contig3\_D2, CL3489.Contig3\_D2, Unigene18573\_D2, Unigene28152\_D2 |
| 50 | Fatty acid metabolism | Unigene14084\_D2, CL331.Contig3\_D2, Unigene26466\_D2, CL3177.Contig1\_D2, CL5475.Contig2\_D2, CL1087.Contig1\_D2, CL2708.Contig2\_D2, CL7143.Contig2\_D2 |
| 51 | Pyruvate metabolism | Unigene21645\_D2, CL51.Contig2\_D2, Unigene20589\_D2, Unigene23072\_D2, CL6804.Contig2\_D2, CL3249.Contig2\_D2, Unigene20806\_D2, CL769.Contig2\_D2, Unigene26042\_D2, CL7633.Contig1\_D2, CL7108.Contig1\_D2, CL6913.Contig1\_D2, Unigene1955\_D2 |
| 52 | Anthocyanin biosynthesis | CL2260.Contig1\_D2, Unigene30431\_D2 |
| 53 | Purine metabolism | CL163.Contig1\_D2, CL678.Contig1\_D2, CL7895.Contig2\_D2, CL7895.Contig1\_D2, CL2569.Contig2\_D2, CL2521.Contig1\_D2, CL124.Contig2\_D2, CL678.Contig2\_D2, Unigene4166\_D2, Unigene7514\_D2, CL1895.Contig1\_D2, CL630.Contig1\_D2, Unigene1782\_D2, Unigene13073\_D2, CL476.Contig1\_D2, CL788.Contig3\_D2, Unigene25049\_D2, Unigene15066\_D2, CL1267.Contig1\_D2, CL5058.Contig1\_D2, CL268.Contig1\_D2, CL207.Contig1\_D2, CL163.Contig3\_D2, Unigene17010\_D2, Unigene17189\_D2, CL7748.Contig1\_D2, CL5892.Contig1\_D2, Unigene19375\_D2, Unigene19446\_D2, Unigene21151\_D2, Unigene28633\_D2, CL7748.Contig7\_D2, CL7889.Contig1\_D2, Unigene284\_D2, CL3502.Contig2\_D2, Unigene1197\_D2, Unigene30469\_D2, Unigene27337\_D2, CL8049.Contig1\_D2, CL7336.Contig1\_D2, Unigene17483\_D2, CL7108.Contig1\_D2, Unigene21362\_D2, Unigene18285\_D2, Unigene29448\_D2 |
| 54 | Regulation of autophagy | CL2503.Contig2\_D2, Unigene29081\_D2, Unigene6290\_D2, CL349.Contig1\_D2, Unigene23640\_D2, Unigene1305\_D2, Unigene21981\_D2, CL3614.Contig2\_D2, Unigene18254\_D2 |
| 55 | Glycerolipid metabolism | Unigene12242\_D2, Unigene21645\_D2, CL51.Contig2\_D2, Unigene19022\_D2, Unigene25801\_D2, Unigene901\_D2, CL6804.Contig2\_D2, CL769.Contig2\_D2, CL6989.Contig2\_D2, Unigene26596\_D2 |
| 56 | Brassinosteroid biosynthesis | Unigene28919\_D2, Unigene17123\_D2, Unigene21945\_D2 |
| 57 | Glycosaminoglycan degradation | Unigene28965\_D2, Unigene21041\_D2, CL1988.Contig3\_D2, Unigene21040\_D2, CL6487.Contig2\_D2 |
| 58 | Monoterpenoid biosynthesis | Unigene18939\_D2, Unigene13449\_D2 |
| 59 | Biotin metabolism | CL2248.Contig1\_D2 |
| 60 | Cysteine and methionine metabolism | CL7935.Contig1\_D2, CL7437.Contig2\_D2, CL7437.Contig1\_D2, CL3178.Contig1\_D2, CL7004.Contig1\_D2, CL562.Contig3\_D2, Unigene4783\_D2, Unigene14113\_D2, CL7199.Contig3\_D2, Unigene18354\_D2, Unigene25931\_D2 |
| 61 | Fructose and mannose metabolism | CL3793.Contig2\_D2, Unigene21645\_D2, Unigene24936\_D2, CL51.Contig2\_D2, Unigene20456\_D2, CL6804.Contig2\_D2, CL769.Contig2\_D2, Unigene19511\_D2, Unigene19512\_D2 |
| 62 | Vitamin B6 metabolism | CL4250.Contig3\_D2, Unigene11272\_D2 |
| 63 | Other types of O-glycan biosynthesis | CL6808.Contig3\_D2, CL5877.Contig1\_D2, CL3630.Contig1\_D2 |
| 64 | Ubiquinone and other terpenoid-quinone biosynthesis | Unigene18442\_D2, Unigene28947\_D2, Unigene22564\_D2, CL5792.Contig1\_D2, CL7199.Contig3\_D2, Unigene373\_D2, CL5792.Contig3\_D2 |
| 65 | C5-Branched dibasic acid metabolism | Unigene30174\_D2 |
| 66 | Propanoate metabolism | CL2130.Contig1\_D2, Unigene20589\_D2, Unigene23072\_D2, Unigene1207\_D2, CL5651.Contig1\_D2, CL6913.Contig1\_D2 |
| 67 | Riboflavin metabolism | Unigene13618\_D2, Unigene31639\_D2, Unigene20412\_D2 |
| 68 | Phenylalanine, tyrosine and tryptophan biosynthesis | Unigene13213\_D2, Unigene4153\_D2, CL7073.Contig2\_D2, Unigene24069\_D2, CL7199.Contig3\_D2, Unigene18354\_D2 |
| 69 | Valine, leucine and isoleucine biosynthesis | Unigene17065\_D2, Unigene24809\_D2, Unigene17066\_D2, Unigene30174\_D2, CL22.Contig5\_D2, Unigene1955\_D2, CL2630.Contig1\_D2 |
| 70 | Arachidonic acid metabolism | Unigene25022\_D2, Unigene30350\_D2 |
| 71 | Spliceosome | CL1331.Contig1\_D2, Unigene1083\_D2, Unigene26813\_D2, Unigene19227\_D2, Unigene17972\_D2, Unigene1727\_D2, CL5655.Contig2\_D2, CL1331.Contig3\_D2, Unigene21501\_D2, CL5029.Contig1\_D2, Unigene22386\_D2, CL283.Contig2\_D2, CL7403.Contig3\_D2, CL1711.Contig2\_D2, CL283.Contig1\_D2, Unigene21502\_D2, CL7403.Contig1\_D2, Unigene1401\_D2, CL5630.Contig1\_D2, CL7403.Contig2\_D2, CL6298.Contig1\_D2, Unigene596\_D2, CL4598.Contig1\_D2, Unigene13479\_D2, Unigene22442\_D2, Unigene19551\_D2, Unigene28364\_D2, CL97.Contig1\_D2, CL6554.Contig3\_D2, Unigene14022\_D2, CL6554.Contig1\_D2, CL6554.Contig2\_D2, Unigene367\_D2, CL1711.Contig1\_D2, CL7167.Contig2\_D2, CL7969.Contig2\_D2, Unigene21326\_D2, CL820.Contig1\_D2, CL7167.Contig1\_D2 |
| 72 | Protein processing in endoplasmic reticulum | CL109.Contig1\_D2, CL1331.Contig1\_D2, Unigene1083\_D2, Unigene27499\_D2, Unigene1727\_D2, CL1331.Contig3\_D2, Unigene30883\_D2, CL283.Contig2\_D2, CL7403.Contig3\_D2, CL283.Contig1\_D2, Unigene15659\_D2, CL7403.Contig1\_D2, Unigene1401\_D2, CL5630.Contig1\_D2, CL7403.Contig2\_D2, Unigene596\_D2, Unigene19068\_D2, CL3715.Contig1\_D2, Unigene1368\_D2, Unigene21151\_D2, Unigene15953\_D2, CL97.Contig2\_D2, CL1876.Contig1\_D2, Unigene8116\_D2, CL1876.Contig2\_D2, CL2586.Contig2\_D2, Unigene24822\_D2, Unigene28132\_D2, Unigene17682\_D2, CL4105.Contig1\_D2 |
| 73 | Sphingolipid metabolism | Unigene28965\_D2, CL1988.Contig3\_D2, CL6487.Contig2\_D2, Unigene901\_D2 |
| 74 | Sesquiterpenoid and triterpenoid biosynthesis | Unigene1584\_D2, CL196.Contig6\_D2 |
| 75 | Carbon fixation in photosynthetic organisms | CL3249.Contig2\_D2, Unigene20806\_D2, Unigene19511\_D2, Unigene19512\_D2, Unigene26042\_D2, CL7108.Contig1\_D2, Unigene18354\_D2 |
| 76 | Thiamine metabolism | Unigene28221\_D2 |
| 77 | beta-Alanine metabolism | CL840.Contig2\_D2, Unigene21137\_D2, Unigene19434\_D2, CL4671.Contig2\_D2 |
| 78 | Protein export | CL109.Contig1\_D2, Unigene1368\_D2, CL1876.Contig1\_D2, CL1876.Contig2\_D2, Unigene23569\_D2 |
| 79 | Peroxisome | CL6522.Contig1\_D2, Unigene26284\_D2, CL507.Contig3\_D2, CL507.Contig2\_D2, CL5475.Contig2\_D2, Unigene30116\_D2, Unigene18238\_D2, CL7143.Contig2\_D2 |
| 80 | Circadian rhythm - plant | Unigene292\_D2, Unigene18967\_D2, Unigene19496\_D2, Unigene4384\_D2, Unigene18751\_D2, Unigene19495\_D2, Unigene300\_D2, Unigene4330\_D2, Unigene20150\_D2, Unigene22441\_D2 |
| 81 | Oxidative phosphorylation | CL6808.Contig3\_D2, Unigene600\_D2, CL400.Contig2\_D2, Unigene20321\_D2, Unigene20320\_D2, CL5202.Contig2\_D2, Unigene18096\_D2, Unigene27461\_D2, CL153.Contig1\_D2, CL8213.Contig1\_D2, CL1035.Contig2\_D2, Unigene24792\_D2, CL1406.Contig2\_D2, Unigene17489\_D2 |
| 82 | Base excision repair | Unigene17313\_D2, Unigene21796\_D2, CL5058.Contig1\_D2, Unigene20770\_D2, Unigene19375\_D2, Unigene21362\_D2, Unigene18285\_D2 |
| 83 | Pantothenate and CoA biosynthesis | Unigene17065\_D2, Unigene24809\_D2, Unigene17066\_D2, Unigene18746\_D2 |
| 84 | Fatty acid elongation | Unigene17419\_D2, Unigene28919\_D2, CL6311.Contig1\_D2 |
| 85 | Pentose phosphate pathway | Unigene24936\_D2, Unigene17189\_D2, Unigene19511\_D2, Unigene19512\_D2, CL844.Contig1\_D2 |
| 86 | Valine, leucine and isoleucine degradation | Unigene17065\_D2, Unigene24809\_D2, Unigene17066\_D2, CL5475.Contig2\_D2, Unigene233\_D2 |
| 87 | Arginine and proline metabolism | CL306.Contig2\_D2, CL4932.Contig1\_D2, Unigene18354\_D2, Unigene20119\_D2, Unigene20120\_D2, Unigene25931\_D2 |
| 88 | Porphyrin and chlorophyll metabolism | Unigene15522\_D2, Unigene24536\_D2, Unigene16827\_D2, Unigene19863\_D2, CL2651.Contig2\_D2, Unigene19815\_D2 |
| 89 | Nucleotide excision repair | CL5058.Contig1\_D2, Unigene19375\_D2, Unigene25107\_D2, CL2362.Contig2\_D2, CL4946.Contig1\_D2, Unigene24822\_D2, Unigene21362\_D2, Unigene18285\_D2 |
| 90 | Phosphatidylinositol signaling system | Unigene12404\_D2, Unigene12405\_D2, Unigene1122\_D2, Unigene22477\_D2, Unigene21161\_D2, Unigene25801\_D2 |
| 91 | Photosynthesis - antenna proteins | CL2303.Contig2\_D2 |
| 92 | Proteasome | CL6593.Contig2\_D2, Unigene15618\_D2, CL5422.Contig2\_D2 |
| 93 | DNA replication | CL5058.Contig1\_D2, Unigene19375\_D2, CL4946.Contig1\_D2, Unigene21362\_D2, Unigene18285\_D2 |
| 94 | Glycosylphosphatidylinositol(GPI)-anchor biosynthesis | Unigene5776\_D2, Unigene16411\_D2, Unigene1612\_D2, CL3984.Contig2\_D2 |
| 95 | Phagosome | Unigene13798\_D2, CL6074.Contig1\_D2, Unigene7313\_D2, Unigene22477\_D2, CL7442.Contig2\_D2, CL1876.Contig1\_D2, CL1876.Contig2\_D2, Unigene18191\_D2 |
| 96 | Sulfur metabolism | CL562.Contig3\_D2 |
| 97 | Glycine, serine and threonine metabolism | CL840.Contig2\_D2, Unigene15618\_D2, Unigene1705\_D2, CL22.Contig5\_D2, Unigene28931\_D2, CL2630.Contig1\_D2 |
| 98 | Steroid biosynthesis | CL2515.Contig3\_D2, Unigene31639\_D2 |
| 99 | Citrate cycle (TCA cycle) | Unigene20806\_D2, Unigene1955\_D2 |
| 100 | Terpenoid backbone biosynthesis | CL8.Contig2\_D2, Unigene25614\_D2, Unigene21335\_D2 |
| 101 | Lysine biosynthesis | CL22.Contig5\_D2 |
| 102 | Inositol phosphate metabolism | Unigene27741\_D2, Unigene22477\_D2, Unigene21161\_D2 |
| 103 | N-Glycan biosynthesis | Unigene24105\_D2 |
| 104 | SNARE interactions in vesicular transport | Unigene19696\_D2 |
| 105 | Natural killer cell mediated cytotoxicity | Unigene13798\_D2 |
| 106 | Basal transcription factors | Unigene16635\_D2, CL2362.Contig2\_D2 |
| 107 | Photosynthesis | Unigene17865\_D2 |
| 108 | Aminoacyl-tRNA biosynthesis | CL1935.Contig1\_D2, CL1631.Contig1\_D2 |
| 109 | Mismatch repair | CL4946.Contig1\_D2 |
| 110 | Homologous recombination | Unigene1575\_D2, CL4946.Contig1\_D2, Unigene12611\_D2, Unigene12612\_D2, CL22.Contig5\_D2, CL2630.Contig1\_D2 |
| 111 | Ribosome | Unigene13465\_D2, CL5999.Contig2\_D2, Unigene28322\_D2, CL3715.Contig1\_D2, Unigene7091\_D2, CL1080.Contig2\_D2, CL2314.Contig3\_D2, CL2315.Contig2\_D2, CL2314.Contig2\_D2, CL5999.Contig1\_D2 |
| 112 | Ubiquitin mediated proteolysis | CL5767.Contig2\_D2, Unigene15659\_D2, Unigene24529\_D2, CL3715.Contig1\_D2, Unigene21730\_D2, Unigene21151\_D2, Unigene13994\_D2, CL4449.Contig2\_D2 |
| 113 | mRNA surveillance pathway | CL6808.Contig3\_D2, CL5993.Contig1\_D2, CL4464.Contig2\_D2, Unigene7514\_D2, Unigene215\_D2, Unigene17239\_D2, CL4808.Contig1\_D2, Unigene10250\_D2, Unigene12666\_D2, Unigene19732\_D2, Unigene24233\_D2, Unigene23520\_D2, CL97.Contig1\_D2, Unigene5047\_D2, Unigene29545\_D2, Unigene15732\_D2, CL3507.Contig2\_D2, Unigene1802\_D2 |
| 114 | RNA transport | Unigene8986\_D2, Unigene26380\_D2, CL6808.Contig3\_D2, CL5993.Contig1\_D2, CL4464.Contig2\_D2, Unigene26813\_D2, Unigene7514\_D2, CL1711.Contig2\_D2, CL7030.Contig2\_D2, CL8168.Contig1\_D2, Unigene215\_D2, Unigene17239\_D2, CL4808.Contig1\_D2, CL6298.Contig1\_D2, Unigene12666\_D2, Unigene19732\_D2, Unigene29812\_D2, CL4470.Contig2\_D2, Unigene19446\_D2, Unigene24233\_D2, CL3528.Contig3\_D2, Unigene9102\_D2, CL97.Contig1\_D2, Unigene5047\_D2, Unigene14022\_D2, Unigene20293\_D2, Unigene1894\_D2, Unigene15732\_D2, Unigene25595\_D2, CL22.Contig5\_D2, CL3507.Contig2\_D2, Unigene1802\_D2, Unigene25345\_D2, Unigene21045\_D2, Unigene21751\_D2, CL2630.Contig1\_D2 |
| 115 | Ribosome biogenesis in eukaryotes | Unigene11707\_D2, Unigene10012\_D2, Unigene6131\_D2, Unigene4384\_D2, Unigene29385\_D2, Unigene215\_D2, CL975.Contig2\_D2, CL586.Contig4\_D2, Unigene11272\_D2, CL810.Contig2\_D2, Unigene17832\_D2 |
| 116 | RNA degradation | Unigene215\_D2, Unigene19732\_D2, Unigene25350\_D2, Unigene19825\_D2, Unigene17832\_D2, Unigene29349\_D2, CL3507.Contig2\_D2, CL3101.Contig2\_D2 |
